# Supplementary material for: Nested non-covalent interactions expand the functions of supramolecular polymer networks
Source: Nat Commun. 2024 May 10;15:3951. doi: 10.1038/s41467-024-47666-x (PMC11087514; doi:10.1038/s41467-024-47666-x)
Supplement: Supplementary file 1 — Supplementary Information [file 41467_2024_47666_MOESM1_ESM.pdf]

Nested Non-covalent Interactions Expand the Functions of Supramolecular Polymer Networks

David J. Lundberg,[1] Christopher M. Brown,[2] Eduard O. Bobylev,[2] Nathan J. Oldenhuis,[3] Yasmeen S. Alfaraj,[2] Julia Zhao,[2] Ilia Kevlishvili,[1] Heather J. Kulik,[1] Jeremiah A. Johnson[2,4]*

[1] Department of Chemical Engineering, Massachusetts Institute of Technology, 77 Massachusetts Avenue, Cambridge, MA 02139, USA

[2] Department of Chemistry, Massachusetts Institute of Technology, 77 Massachusetts Avenue, Cambridge, MA 02139, USA

[3] Department of Chemistry, University of New Hampshire, 23 Academic Way, Durham, NH 03824, USA

[4] David H. Koch Institute for Integrative Cancer Research, Massachusetts Institute of Technology, 77 Massachusetts Avenue, Cambridge, Massachusetts 02139, USA

Supplementary Note 1: Guest Binding Studies **3**

HSO_4 in MOC 3

HSO_4 in N-MOC 4

NO_3 in MOC 5

NO_3 in N-MOC 6

DAQ in MOC 7

DAQ in N-MOC 9

Supplementary Note 2: MOC Characterization **10**

NO_3⊂MOC Characterization 12

HSO_4⊂MOC Characterization 14

DAQ⊂MOC Characterization 16

Supplementary Note 3: Synthesis and Characterization of Compounds, MOCs, and PolyMOCs **18**

Ethyl-3,5-dibromobenzoate 18

L1 21

L1 Acid 24

Polymer Ligand (PL) 27

L2 29

MOC Synthesis - With Added Guest 32

MOC Synthesis - With Excess Palladium 32

PolyMOC Fabrication with Added Guest 32

Excess Palladium polyMOC Gel Synthesis 32
Additional Small-molecule Ligand polyMOC Gel Synthesis 33
^{1}H NMR CP-MAS Characterization of PolyMOC Gels 34
Representative Frequency Sweep Data for PolyMOC Gels 38
Stress Relaxation Modeling 39
Modeling of Excess Pd^{2+} and Mixed-Ligand PolyMOC Network Structures 40
Characterization of Gels Fabricated with Excess Pd^{2+} 43
Guest-Triggered Sol-Gel Transitions 48
Guest Uptake into Preformed PolyMOC Gels 49
High-Resolution Mass-Spectrometry Characterization of MOCs 50
Single Crystal X-Ray Diffraction Studies 54
Crystal Growth Conditions 54
Crystallography Methods 54
Crystal Structure Analysis 55
Thermal Ellipsoid Plots of Crystal Structures 59
Supplementary Note DFT Calculations........ 63
Supplementary References........ 63

Supplementary Note 1: Guest Binding Studies

HSO_4 in MOC

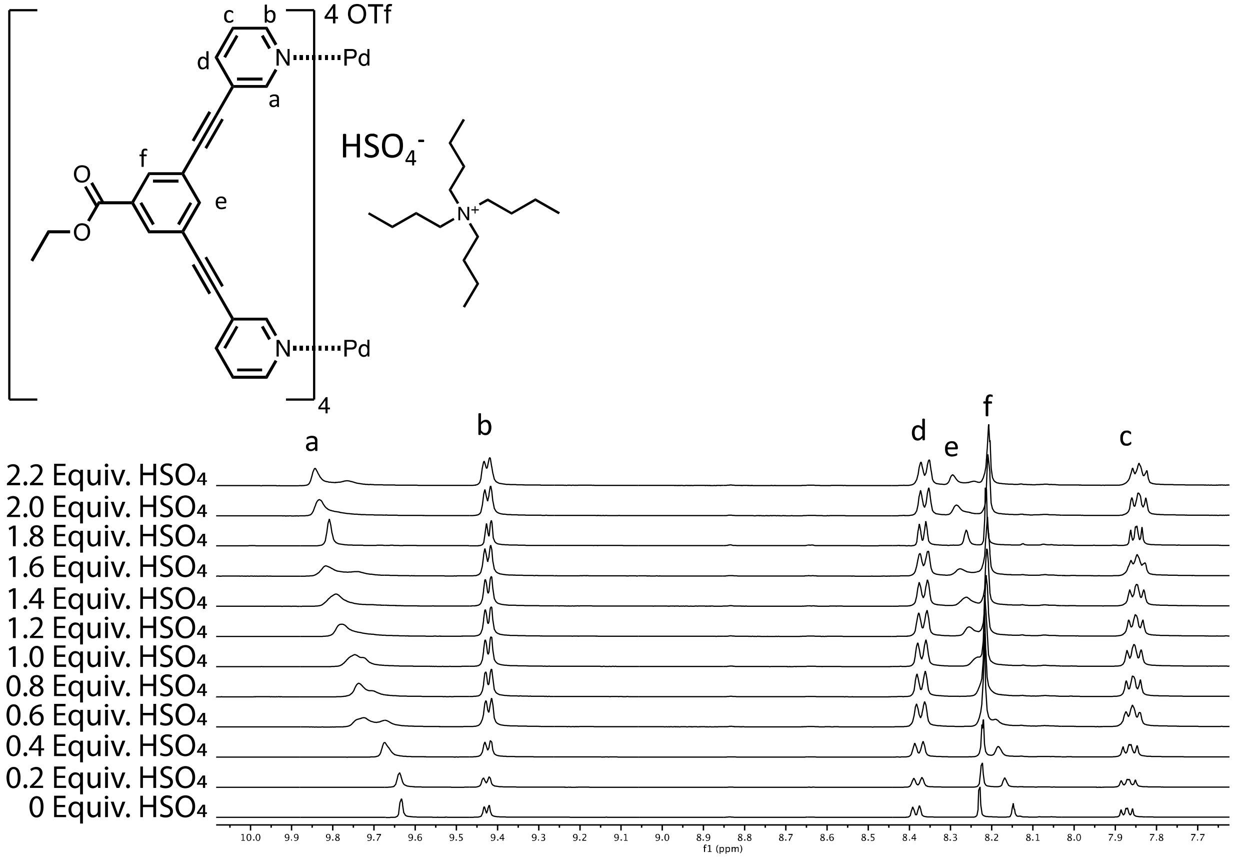

Supplementary Figure S1. ^{1}H NMR spectra of **HSO_4** titration into **MOC**. The chemical shifts of peaks b, c, d, e, and f were fit to a 1:1 binding isotherm to measure the association constant. Note, the varying broadness of the most downfield peak precluded its inclusion in titration isotherm fitting to determine the association constant. The association constant was measured to be **K_a = 1000 ± 200 M^{-1}**.

HSO_4 in N-MOC

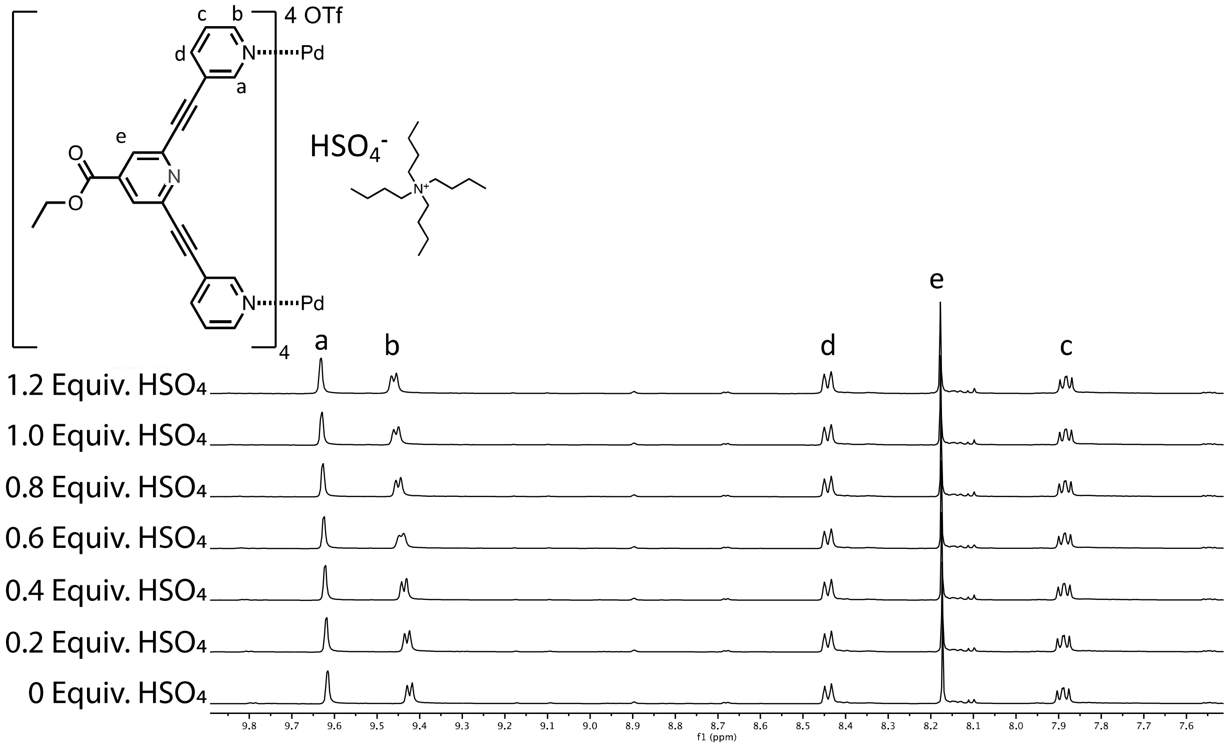

Supplementary Figure S2. ^{1}H NMR spectra of **HSO_4** titration into **N-MOC**. The chemical shifts of peak a were fit to a 1:1 binding isotherm to measure the association constant of **K_a = 33 ± 10 M^{-1}**.

NO_3 in MOC

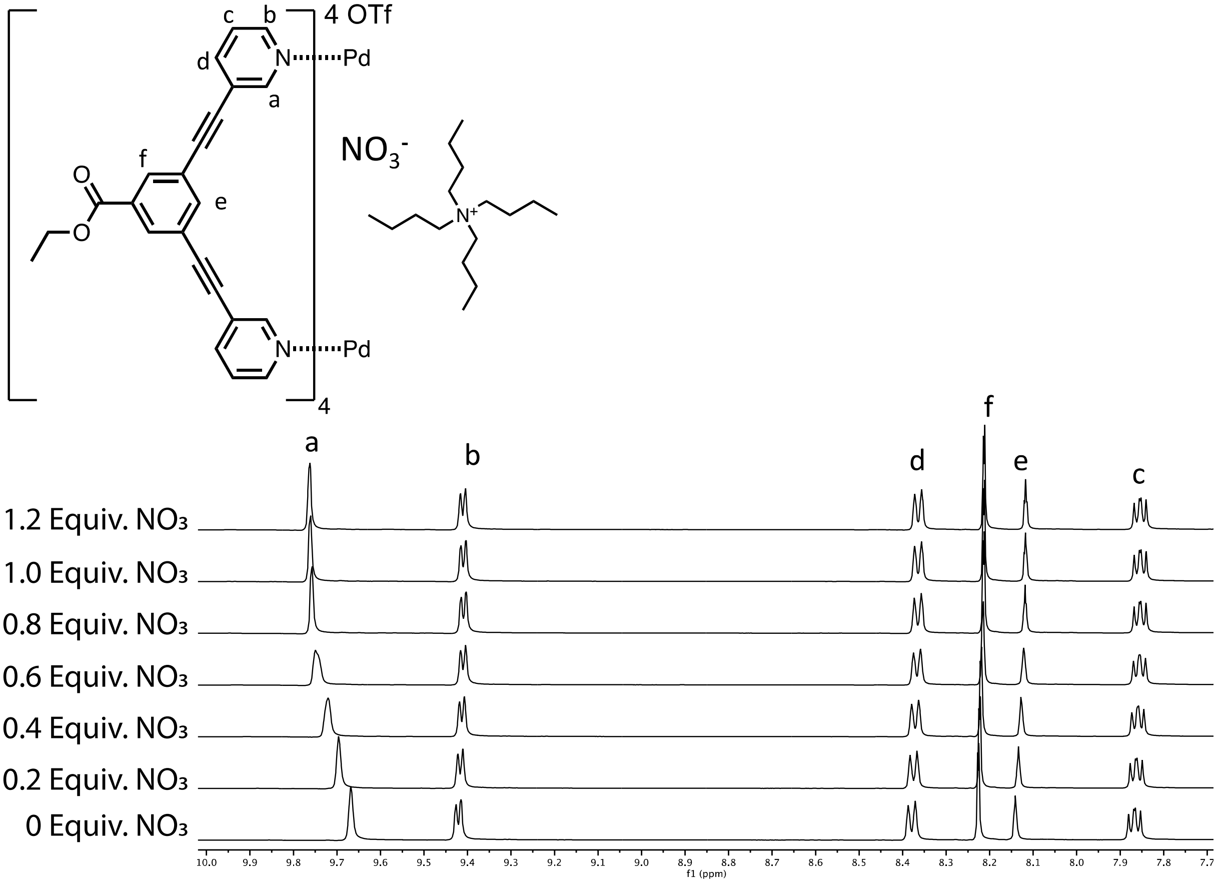

Supplementary Figure S3. ^{1}H NMR spectra of **NO_3** titration into **MOC**. The chemical shifts of peaks a, b, d, e were fit to a 1:1 binding isotherm to measure the association constant of **K_a = 6,000 ± 1,000 M^{-1}**.

NO_3 in N-MOC

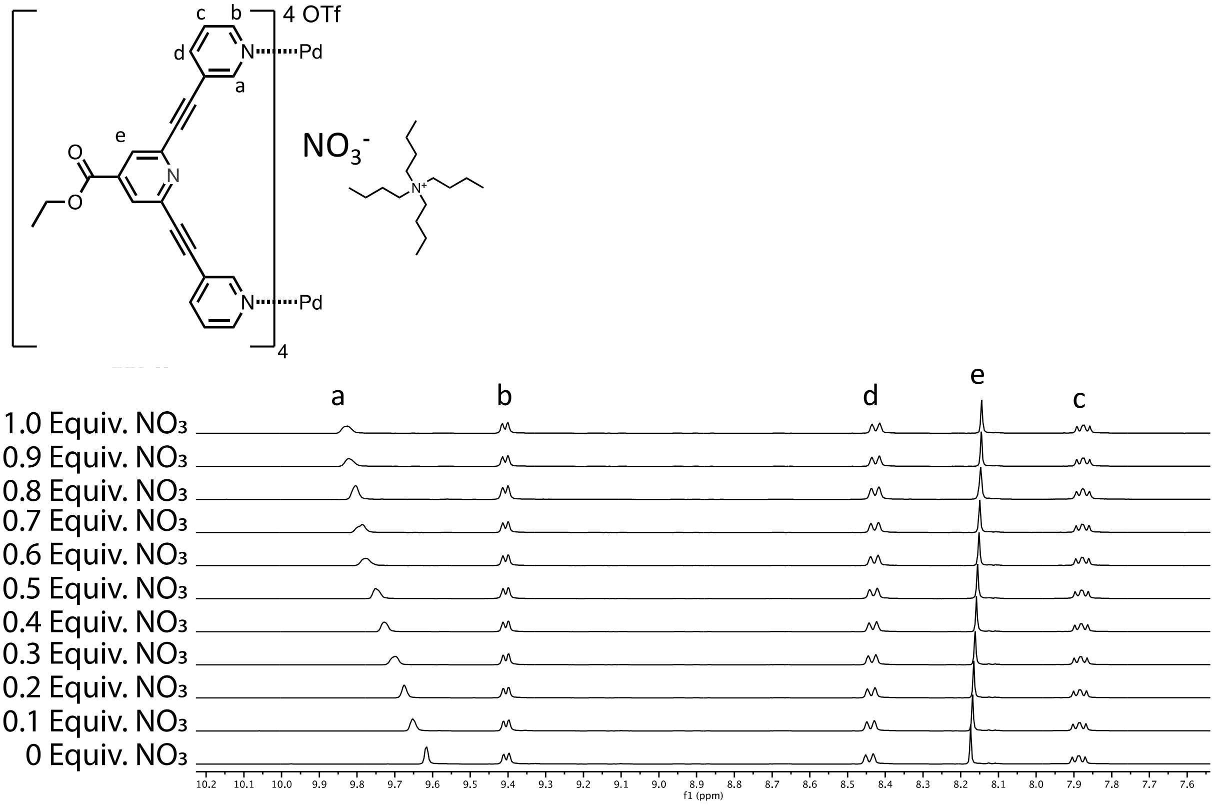

Supplementary Figure S4. ^{1}H NMR spectra of **NO_3** titration into **N-MOC**. The chemical shifts of peaks a, d and e were fit to a 1:1 binding isotherm to measure the association constant of **K_a = 2,200 ± 400 M^{-1}**.

DAQ in MOC

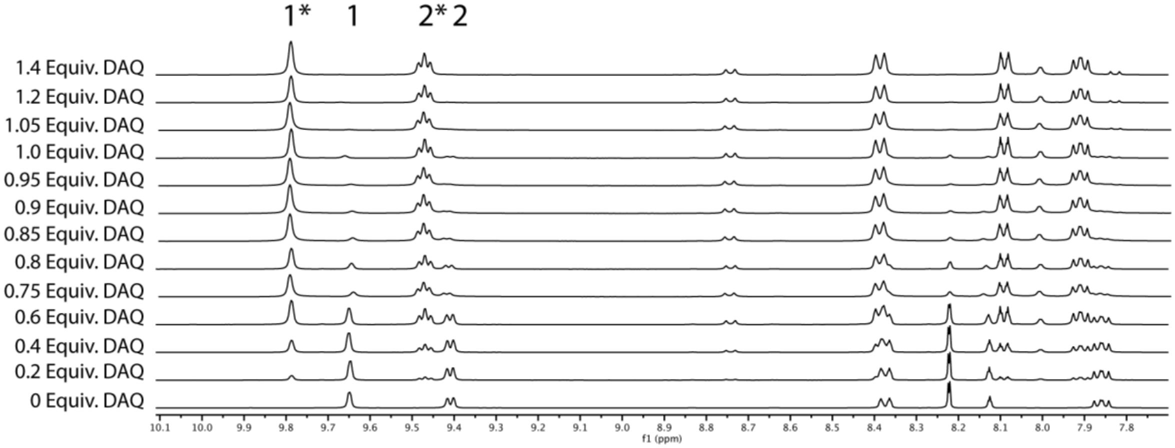

Supplementary Figure S5. ^{1}H NMR Titration data for **DAQ** in **MOC**. The resonances 1 and 2 belong to **MOC**, and the resonances labeled 1* and 2* belong to **DAQ⊂MOC**. Comparison of their relative integrations was used to calculate the association constant (see Supplementary Figure S6 below).

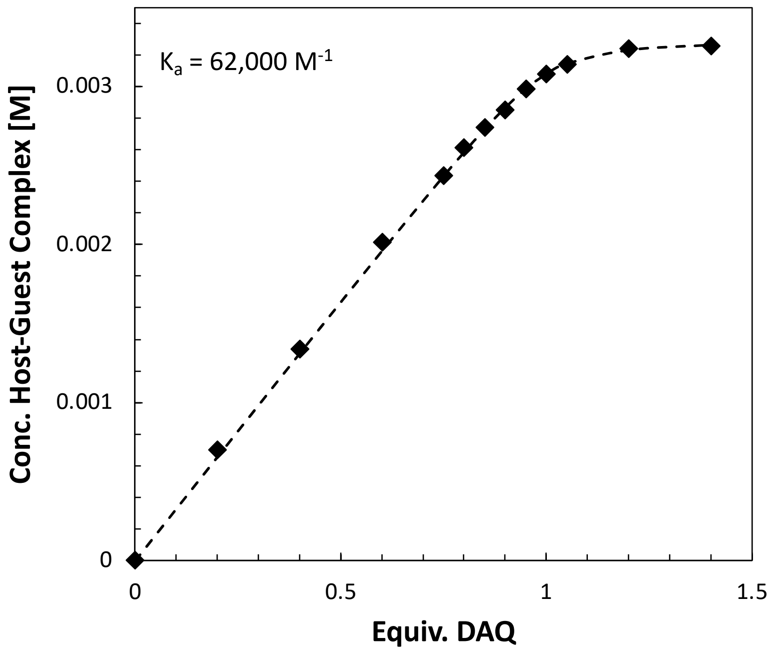

Supplementary Figure S6. Observed and fit host-guest complex concentration data used to calculate the association constant for **DAQ.**

The association constant for this system was fit by calculating the concentration of host and guest-bound host molecules by comparing the relative integrations between peaks 1/1* and 2/2* and taking the average. These data were then fit to the expression:

$$K_a = \frac{[HG]}{[H][G]}$$

Where [HG], [H], and [G] are the concentrations of host-guest complex, unbound host, and unbound guest, respectively.

DAQ in N-MOC

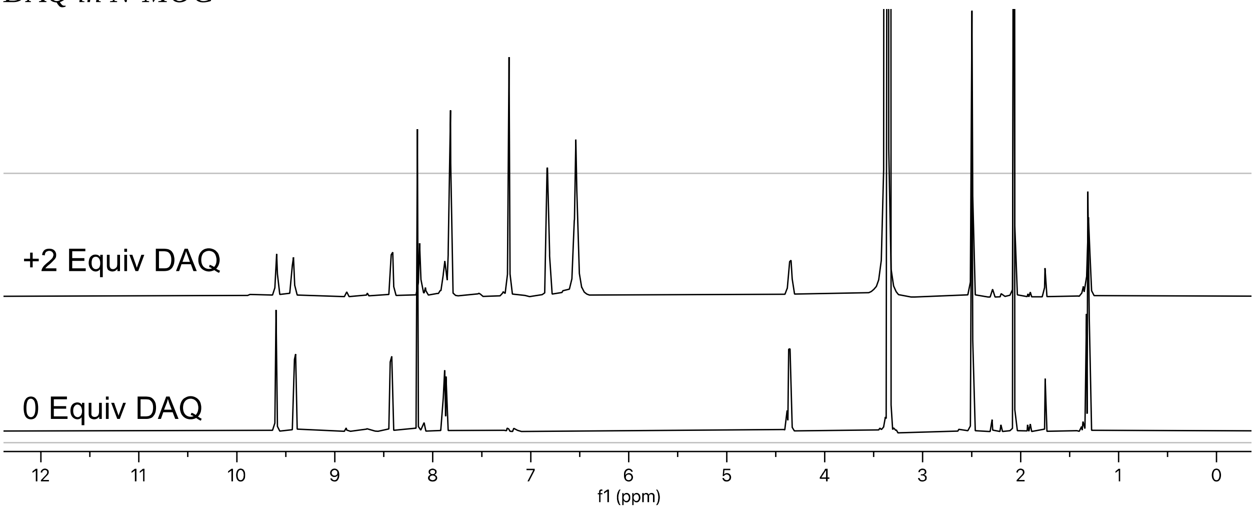

Supplementary Figure S7. ^{1}H NMR Spectrum of **N-MOC** with 0 or 2 equiv. of **DAQ**. No significant peak shifts are observed upon guest addition, indicating relatively weak or no guest binding in this system.

Supplementary Note 2: MOC Characterization

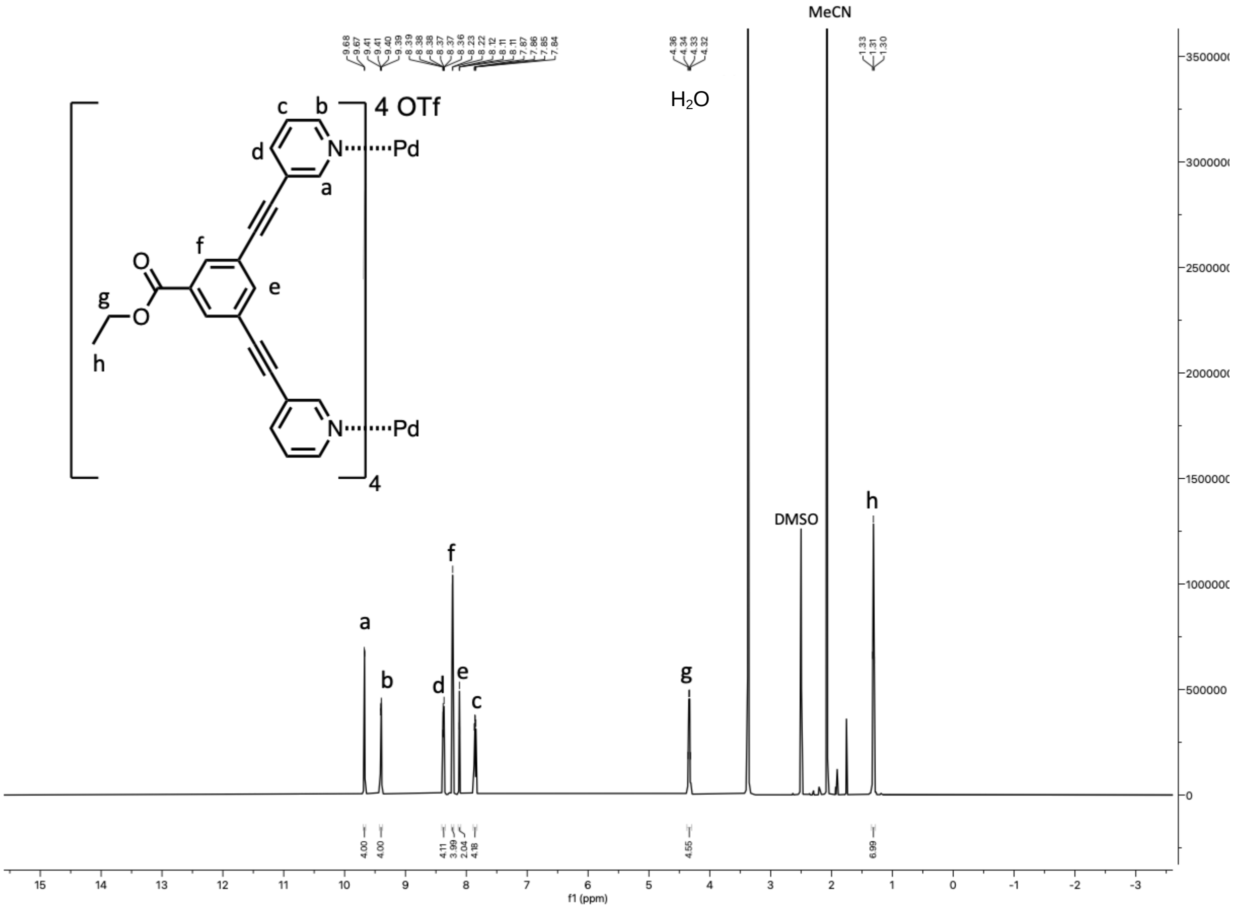

Supplementary Figure S8. ^{1}H NMR (500 MHz, 25 °C, DMSO-d_6) of **MOC**.

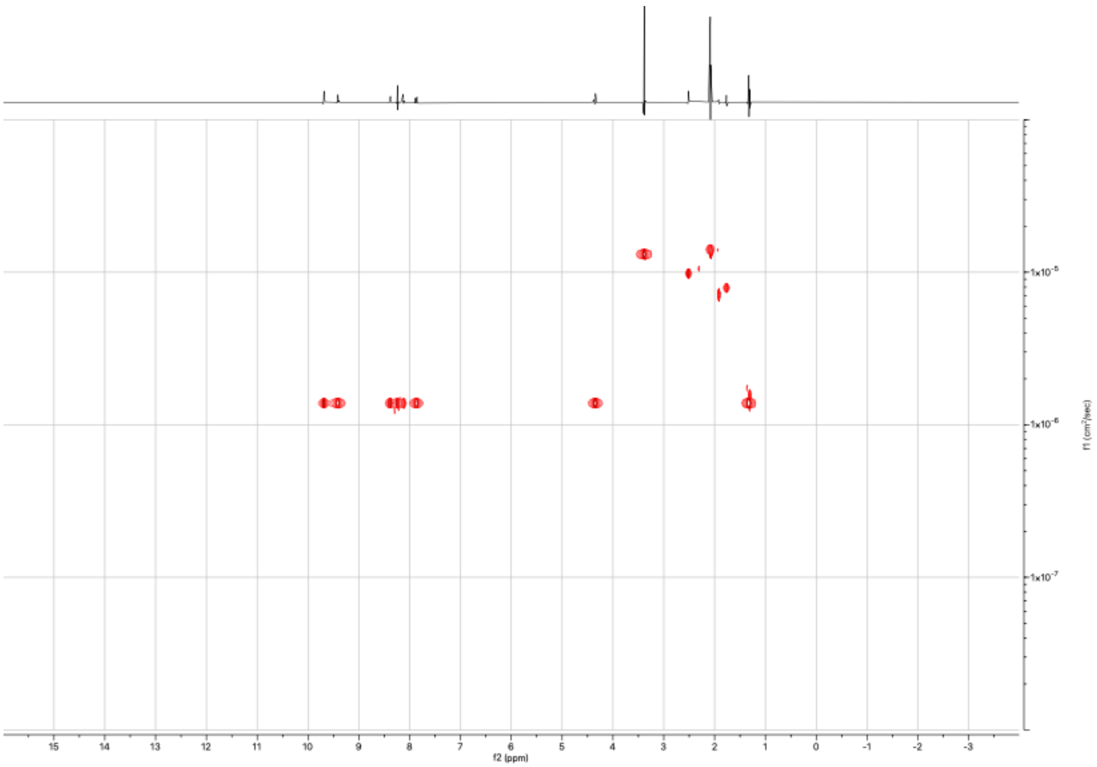

Supplementary Figure S9. ^{1}H DOSY NMR (500 MHz, 25 °C, DMSO-d$_6$) of **MOC**. The diffusion constant of the MOC was measured to be 1.35×10^{-6} cm^2/s which corresponds to a hydrodynamic radius of 8.13×10^{-10} m (diameter of 1.62 nm).

NO_3⊂MOC Characterization

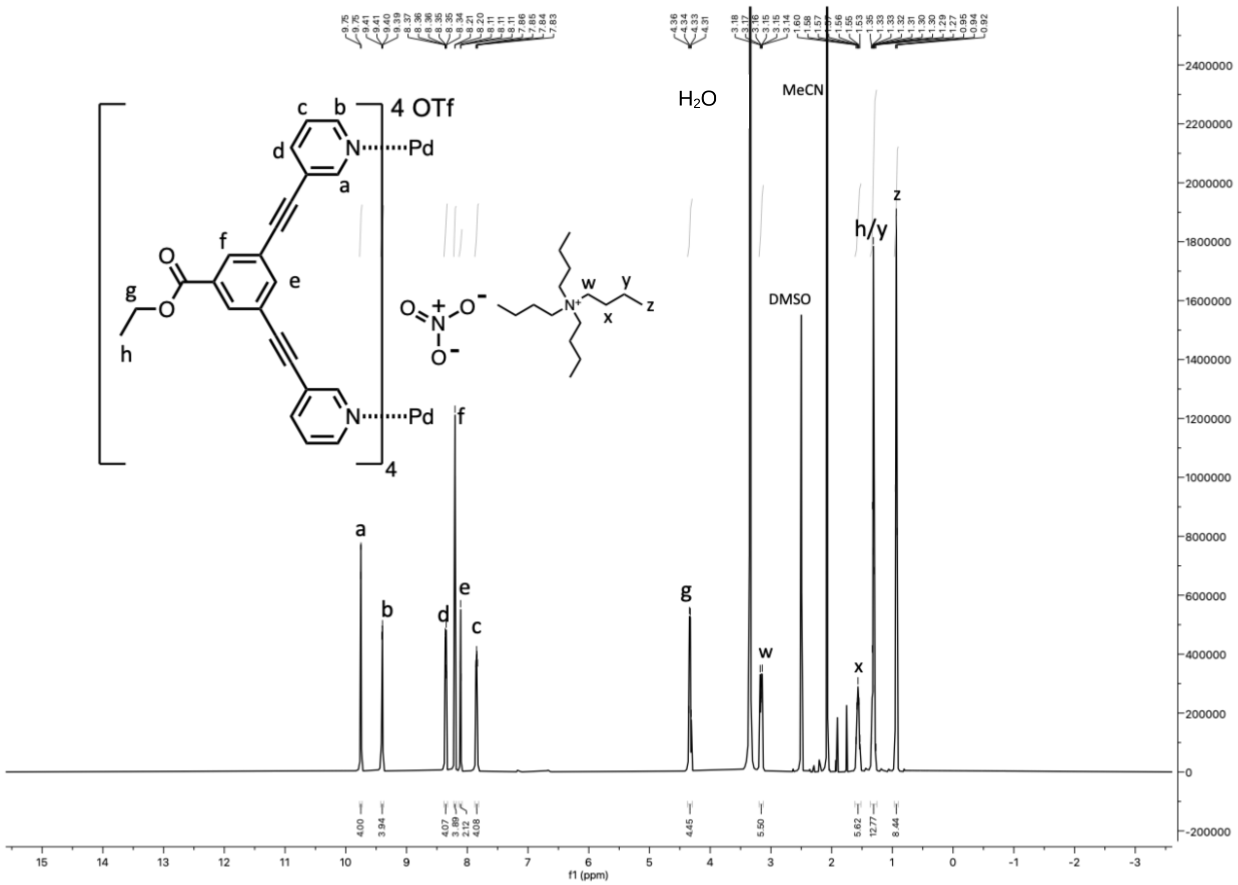

Supplementary Figure S10. ^{1}H NMR (500 MHz, 25 °C, DMSO-d_6) of **NO_3⊂MOC.**

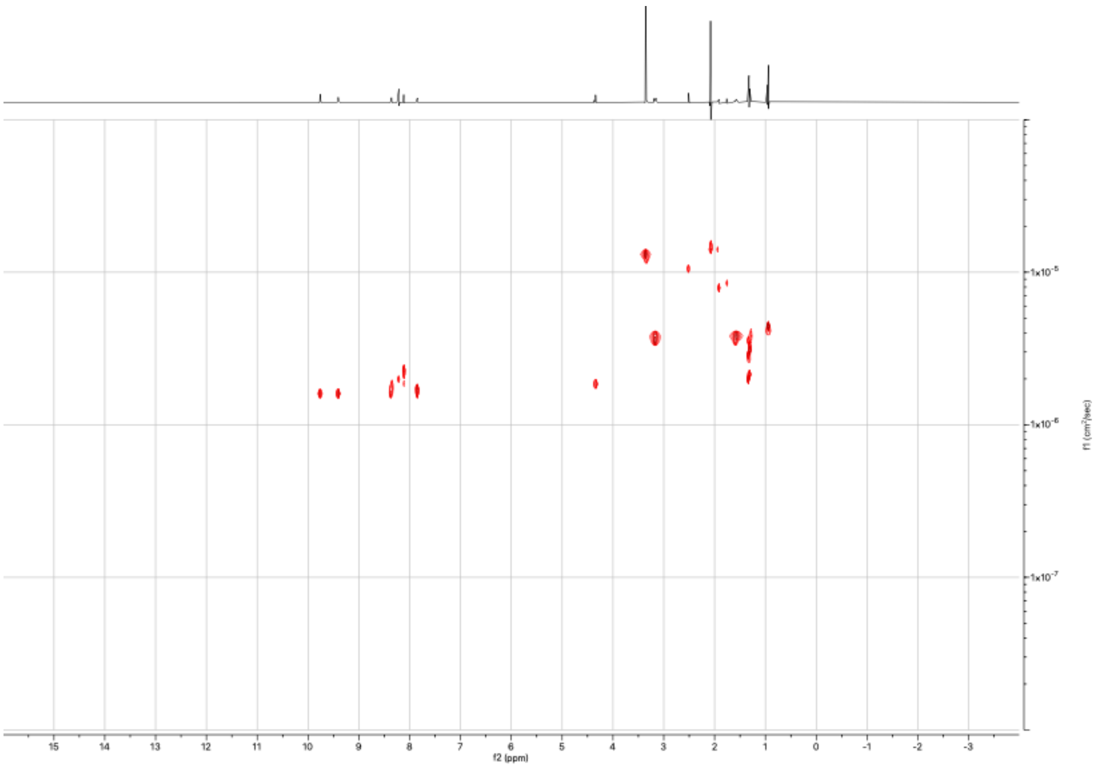

Supplementary Figure S11. ^{1}H DOSY NMR (500 MHz, 25 °C, DMSO-d_6) of **NO_3⊂MOC**. The diffusion constant of the MOC was measured to be 1.42×10^{-6} cm^2/s which corresponds to a hydrodynamic radius of 7.73×10^{-10} m (diameter of 1.55 nm).

HSO_4⊂MOC Characterization

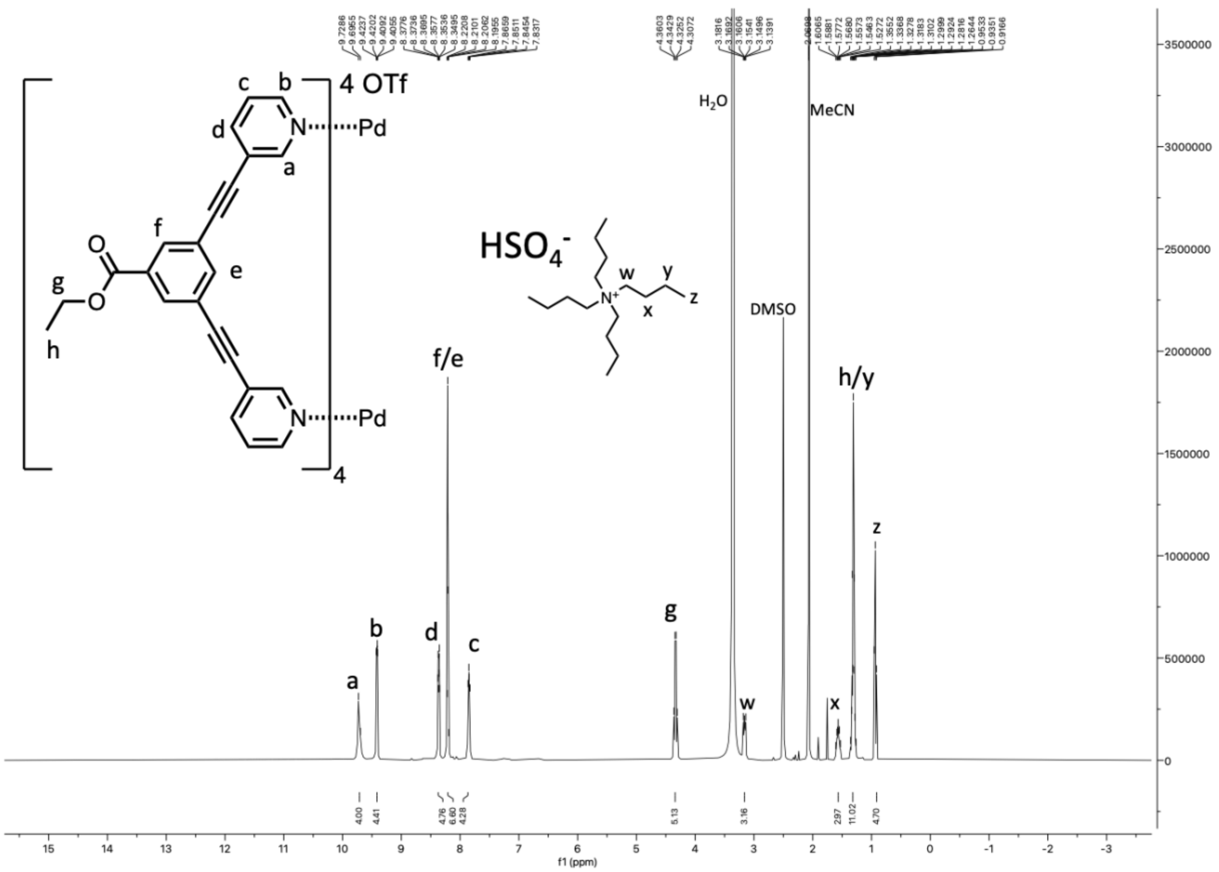

Supplementary Figure S12. ^{1}H NMR (500 MHz, 25 °C, DMSO-d_6) of **HSO_4⊂MOC.**

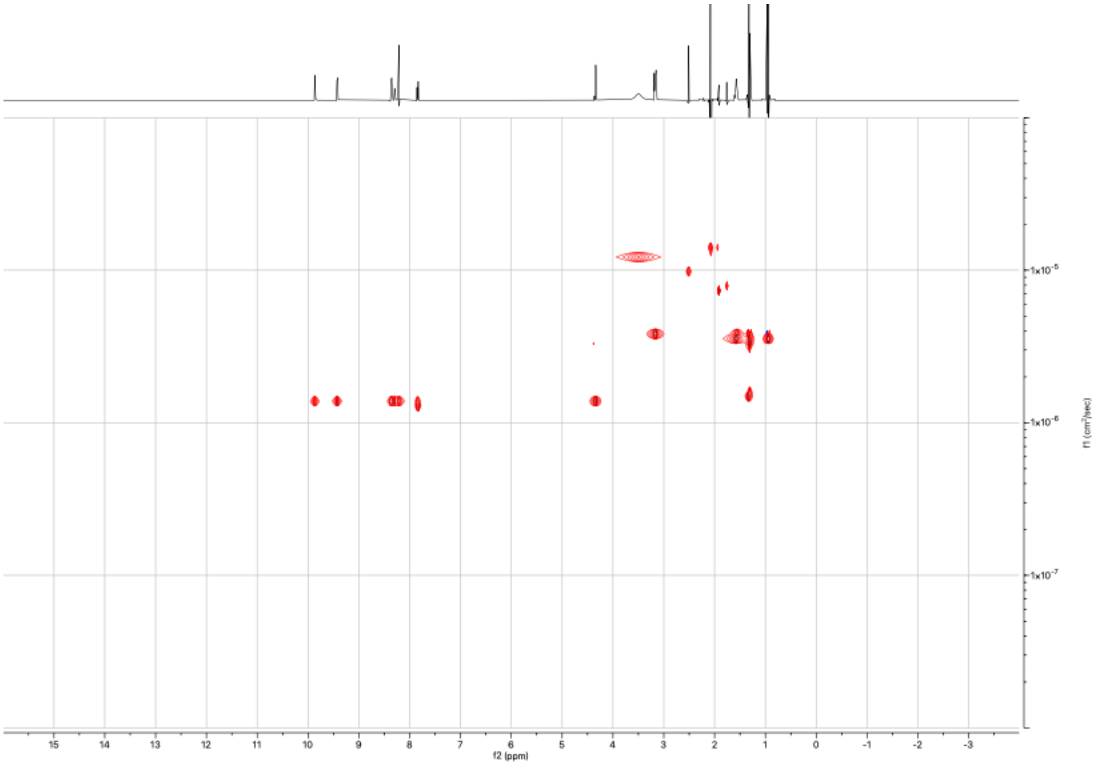

Supplementary Figure S13. ^{1}H DOSY NMR (500 MHz, 25 °C, DMSO-d_6) of **HSO_4⊂MOC**. The diffusion constant of the MOC was measured to be 1.40×10^{-6} cm^2/s which corresponds to a hydrodynamic radius of 7.78×10^{-10} m (diameter of 1.57 nm).

DAQ⊂MOC Characterization

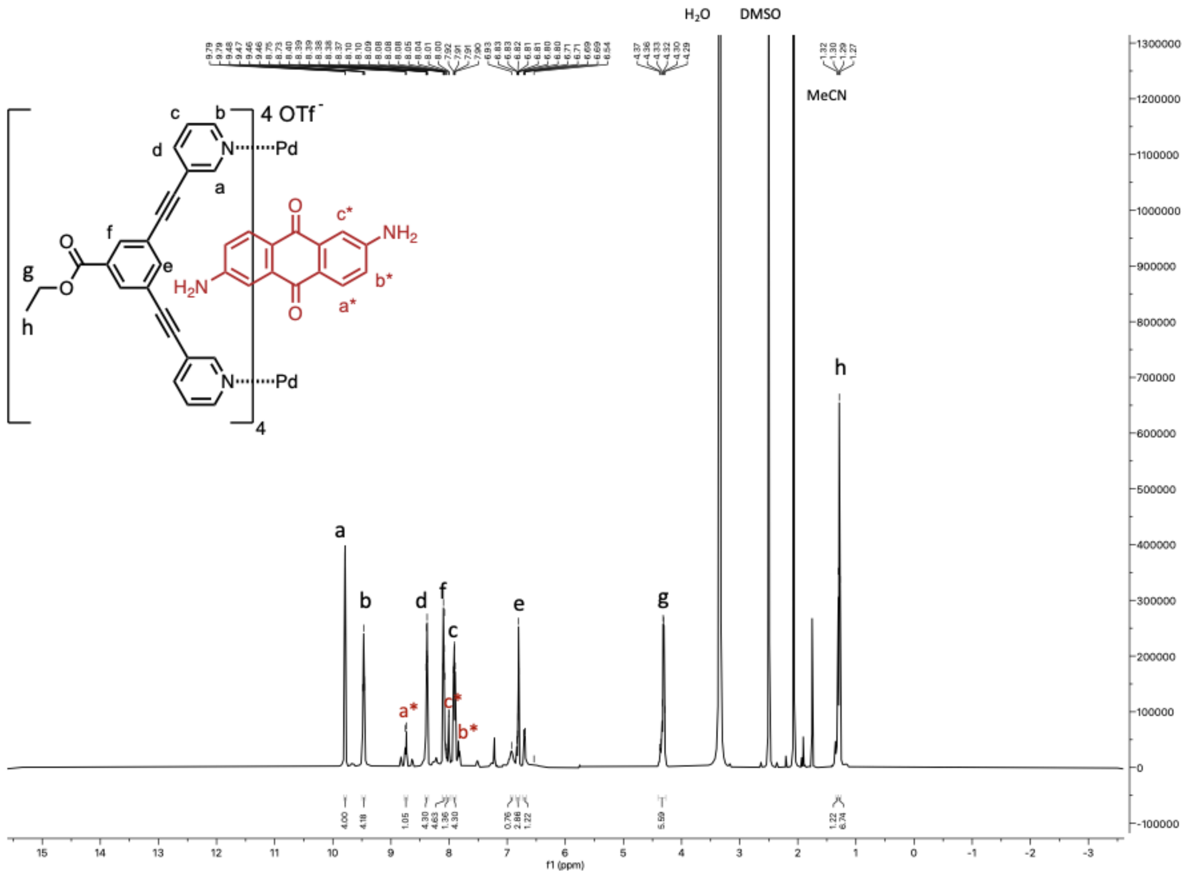

Supplementary Figure S14. ^{1}H NMR (500 MHz, 25 °C, DMSO-d$_6$) of **DAQ⊂MOC.**

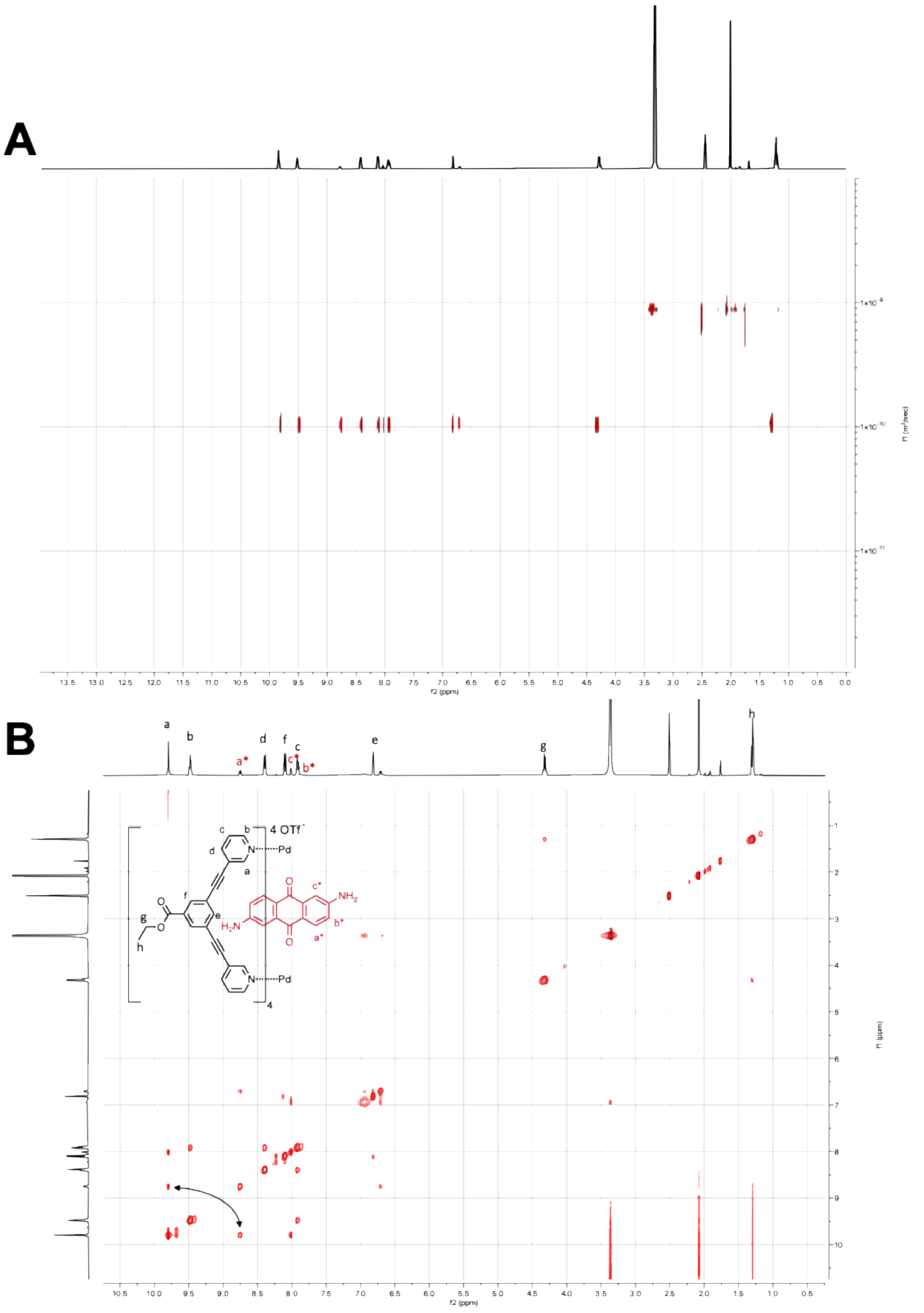

Supplementary Figure S15. A. ^{1}H DOSY NMR (500 MHz, 25 °C, DMSO-d$_6$) of **DAQ⊂MOC**. The diffusion constant of the MOC was measured to be 1.13×10^{-6} cm^2/s which corresponds to a hydrodynamic radius of 9.97×10^{-10} m (diameter of 1.93 nm). Resonances of **DAQ** are on the same band as those associated with the metal-organic cage structure. **B.** ^{1}H NOESY NMR (500 MHz, 25 °C, DMSO-d$_6$) of **DAQ⊂MOC.** Coupling between resonances associated with the interior of the MOC (H$_a$) and **DAQ** (H$_{a*}$) is observed and denoted by an arrow.

Supplementary Note 3: Synthesis and Characterization of Compounds, MOCs, and PolyMOCs

Ethyl-3,5-dibromobenzoate

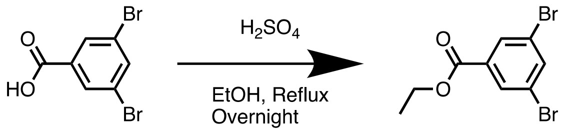

2,5-Dibromobenzoic acid (15 g, 54 mmol, 1 equiv.) and H_2SO_4 (5 mL,) were added to 300 mL absolute ethanol and heated to reflux overnight (16 hours). The reaction mixture was cooled to room temperature and ethanol was removed via rotary evaporator. The dry mixture was diluted with 250 mL of ethyl acetate and washed twice with 250 mL of saturated aqueous sodium bicarbonate solution and once with 250 mL of brine. After washing, the organic layer was dried over Na_2SO_4, filtered, and dried *in vacuo*. 11 g of a pale-yellow solid was obtained. 96% isolated yield.

^{1}H NMR (500 MHz, $CDCl_3$): δ 8.10 (s, 2H), 7.84 (s, 1H), 4.39 (q, J = 7.1 Hz, 2H), 1.40 (t, J = 7.1 Hz, 3H).

^{13}C{^{1}H} NMR (151 MHz, $CDCl_3$): δ 164.2, 138.3, 133.8, 131.5, 123.1, 62.0, 14.4

DART-HRMS (m/z) calculated for ($C_9H_9Br_2O_2$) = 307.8871 $[M+H]^+$; found 307.8867

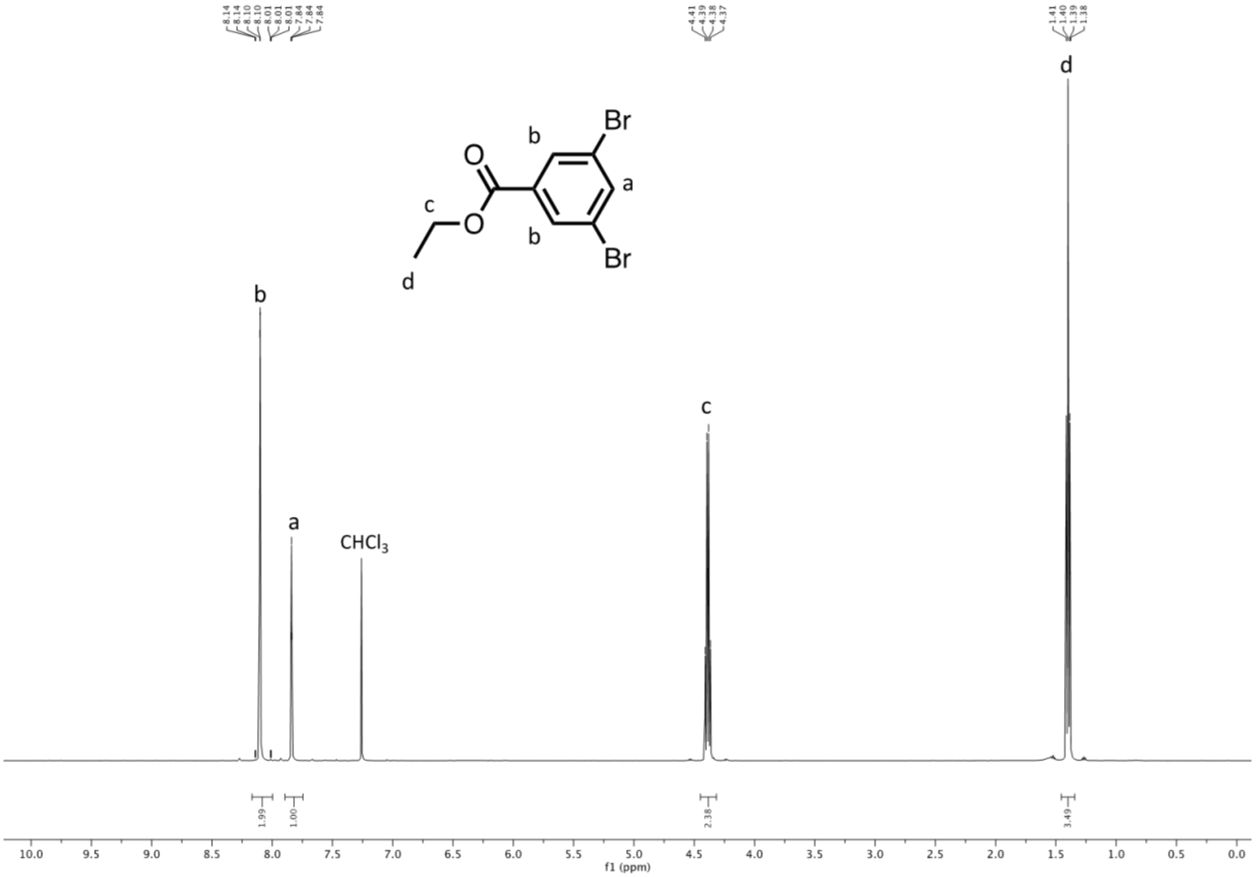

Supplementary Figure S16. ^{1}H NMR (500 MHz, 25 °C, $CDCl_3$) Ethyl-3,5-bromobenzoate

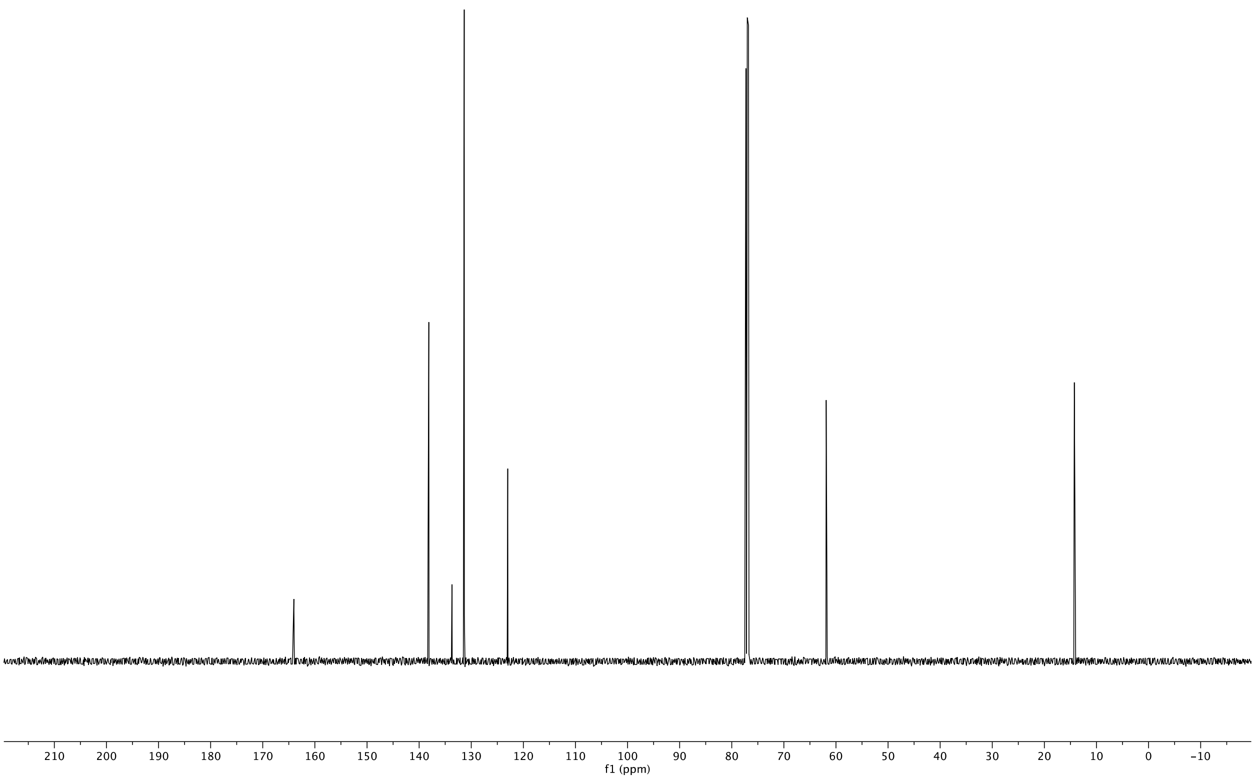

Supplementary Figure S17. $^{13}C\{^{1}H\}$ NMR (151 MHz, 25 °C, $CDCl_3$) spectra of ethyl-3,5-bromobenzoate.

L1

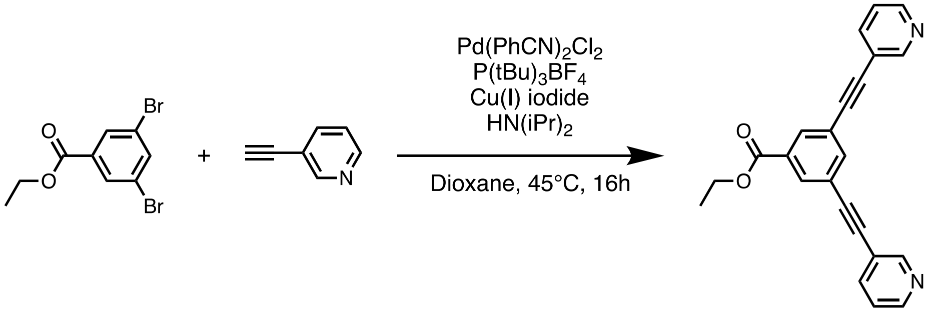

Ethyl-3,5-dibromobenzoate (5.0 g, 16.2 mmol, 1 equiv.), 3-ethyylpyridine (4.3 g, 40.5 mmol, 2.5 equiv.), copper(I) iodide (117 mg, 0.61 mmol, 0.036 equiv.), and tri-tert-butylphosphonium tetrafluoroborate (704 mg, 2.43 mmol, 0.15 equiv.) were added to a round bottom flask and which was sealed and flushed with nitrogen 3 times. 200 mL of anhydrous dioxane was added via cannula into the reaction flask, followed by 20 mL of diisopropylamine. The reaction mixture was sparged with nitrogen for 60 minutes before bis(benzonitrile)palladium(II) dichloride (466 mg, 1.22 mmol, .075 equiv.) was added under positive nitrogen pressure. The reaction mixture was further spared with nitrogen for 15 minutes and let stir at 45°C for 16 hours before being cooled to room temperature and filtered through Celite and washed with ethyl acetate until the filtrate was colorless. The resulting dioxane/ethyl acetate solution (~500 mL) was washed with 10% aqueous ethylene diamine (250 mL × 2) and brine (500 mL × 1) before drying the organic layer with Na_2SO_4, filtering, and concentrating to dryness. The crude product was purified using silica column chromatography (0-8% MeOH in DCM gradient, product eluting at 4% MeOH). 4.2 g of a pale-yellow solid was obtained (73% isolated yield). For experiments involving MOC assembly in solution, the product was further purified via recrystallization from boiling toluene.

^{1}H NMR (500 MHz, $CDCl_3$): δ 8.79 (s, 2H), 8.61 (d, *J* = 4.9, 1.7 Hz, 2H), 8.19 (s, 2H), 7.89 (s, 1H), 7.85 (dt, *J* = 7.9, 1.9 Hz, 2H), 7.33 (ddd, *J* = 7.9, 5.0, 1.0 Hz, 2H), 4.45 (q, *J* = 7.2 Hz, 2H), 1.46 (t, *J* = 7.1 Hz, 3H)

$^{13}C\{^{1}H\}$ NMR (151 MHz, $CDCl_3$): δ 165.2, 152.4, 149.1, 138.8, 138.3, 132.8, 131.6, 123.7, 123.3, 120.0, 90.9, 87.6, 61.8, 14.5.

DART-HRMS (m/z) calculated for ($C_{23}H_{16}N_2O_2$) = 353.1312 $[M+H]^+$; found 353.1316

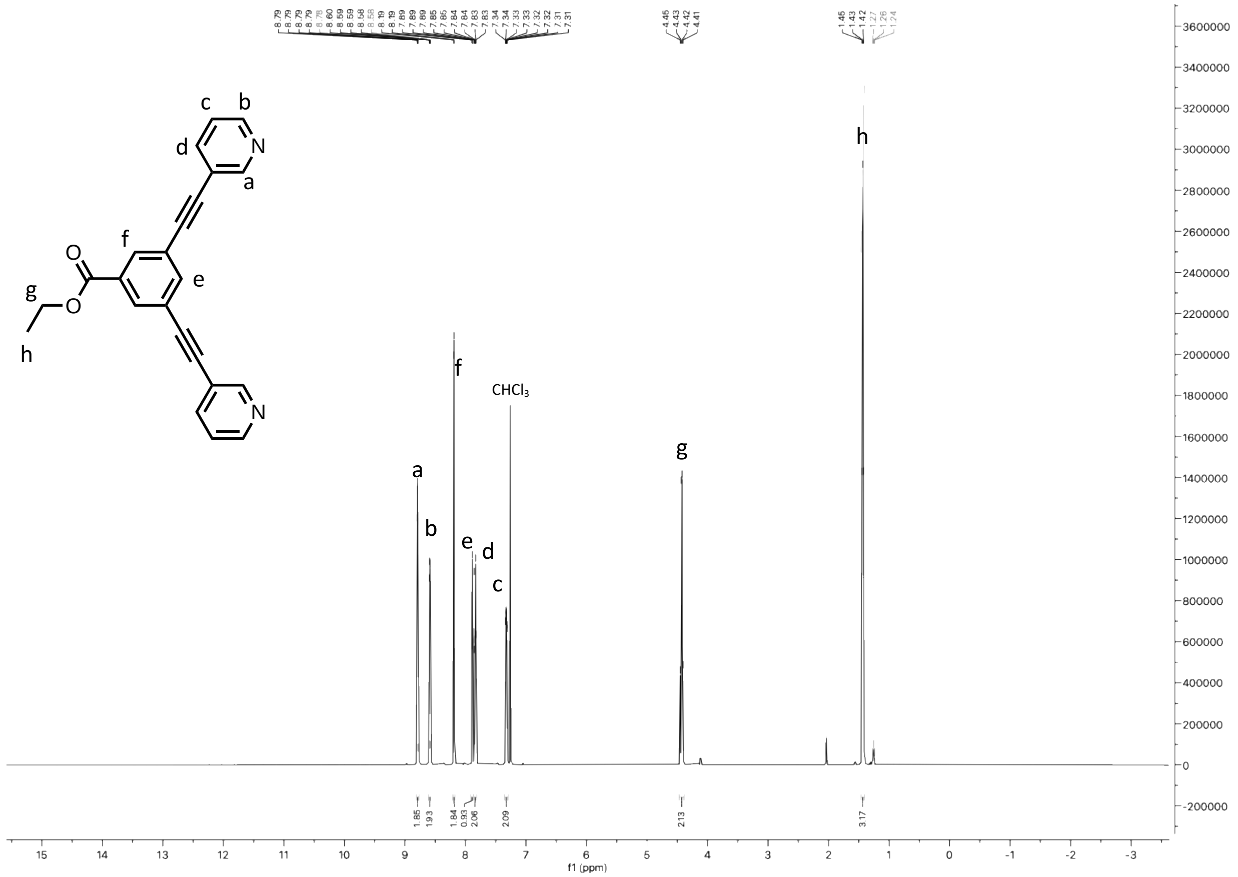

Supplementary Figure S18. ^{1}H NMR (500 MHz, 25 °C, $CDCl_3$) spectrum of **L1**.

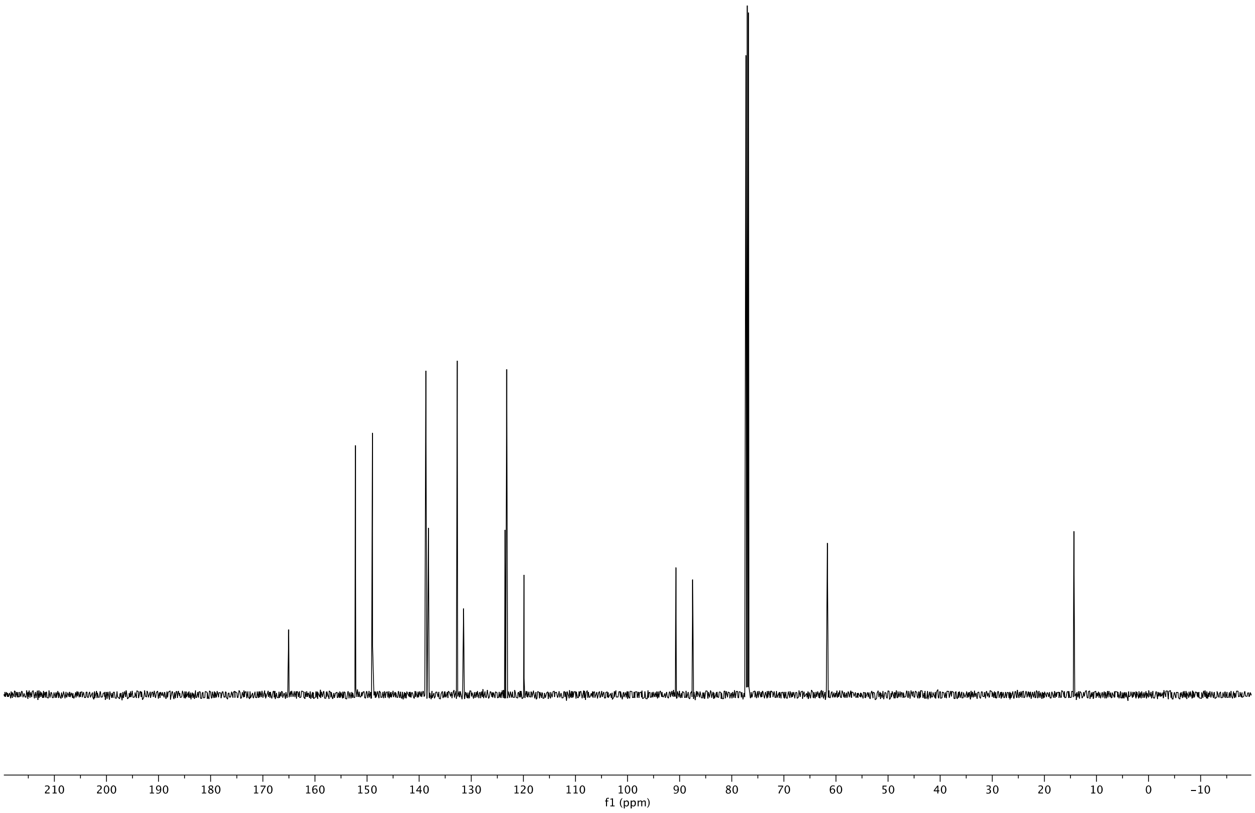

Supplementary Figure S19. $^{13}C\{^{1}H\}$ NMR (151 MHz, 25 °C, $CDCl_3$) spectrum of **L1**.

L1 Acid

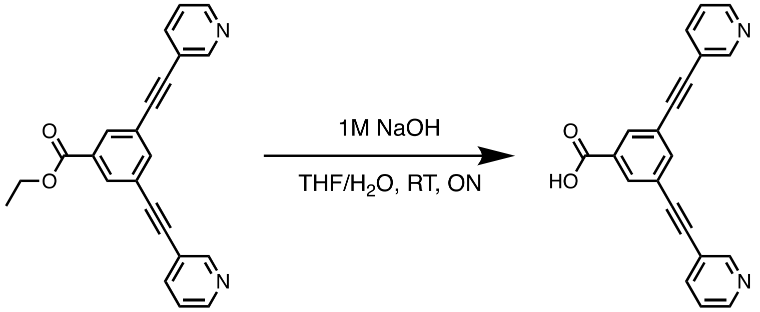

Bis-ligand ester (2g, 5.7 mmol) was dissolved in 10 mL of 50/50 THF/H_2O to which 2 mL of 1M aqueous NaOH solution was added before the reaction mixture was left to stir at room temperature overnight. Afterward, THF was removed via rotary evaporation and the aqueous mixture dissolved in ~500 mL H_2O. The pH of this solution was adjusted to ~4.5 through dropwise addition of 1M aqueous HCl, upon which the product precipitated. The precipitate was collected through filtration and centrifugation and thoroughly dried under vacuum at 50°C. 1.6g of a colorless solid was obtained. 86% isolated yield.

^{1}H NMR (500 MHz, DMSO-d_6): δ 8.89 (m, 2H), 8.68 (dd, *J* = 5.1, 2.0 Hz, 2H), 8.15 (d, *J* = 1.6 Hz, 4H), 8.08 (t, *J* = 1.7 Hz, 1H), 7.60 (dd, *J* = 7.7, 4.7 Hz, 2H).

^{13}C{^{1}H} NMR (151 MHz, DMSO-d_6 / THF-d_8): δ 168.83, 151.37, 148.96, 140.45, 139.10, 134.76, 132.99, 124.10, 121.99, 119.85, 91.6, 86.2.

DART-HRMS (m/z) calculated for ($C_{21}H_{12}N_2O_2$) = 325.0999 $[M+H]^+$; found 325.1009

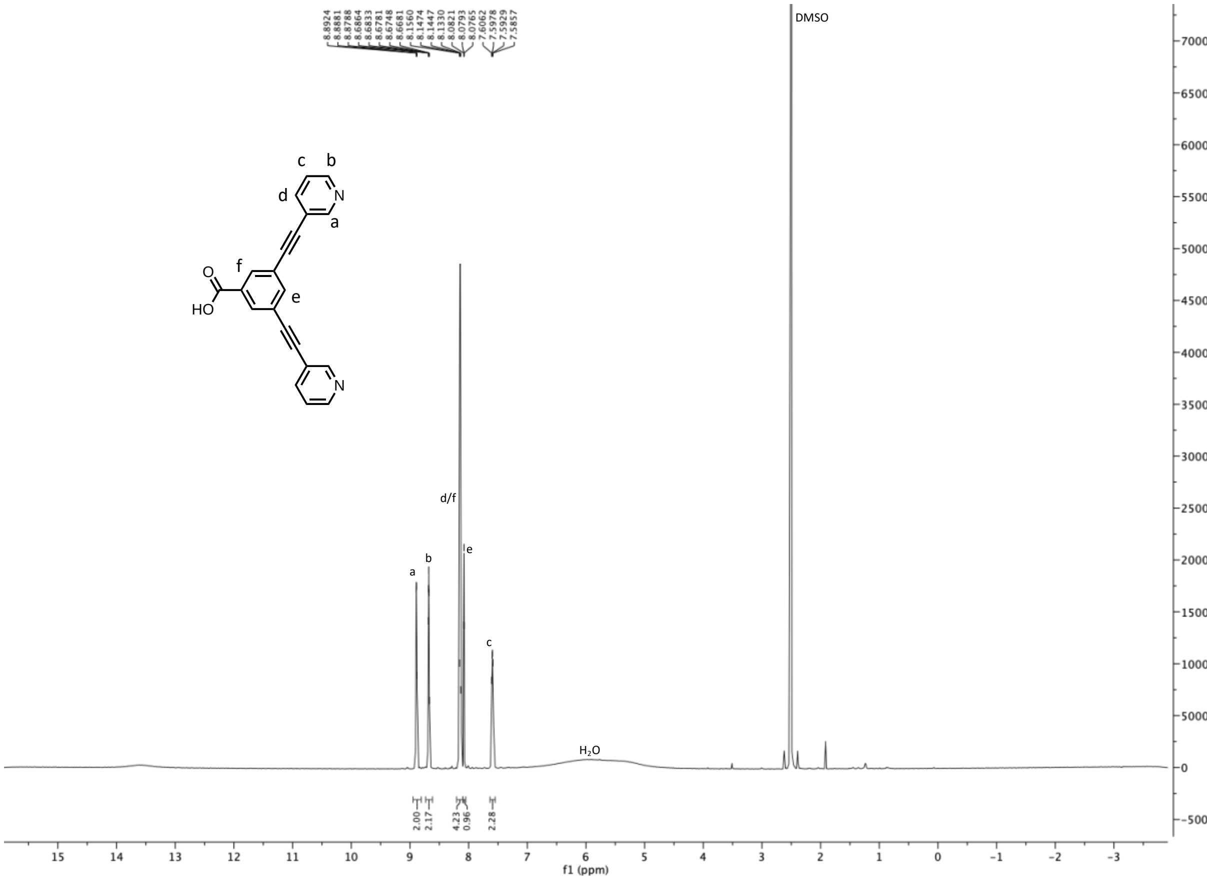

Supplementary Figure S**20.** ^{1}H NMR (500 MHz, 25 °C, DMSO-d$_6$) spectrum of **L1 Acid**.

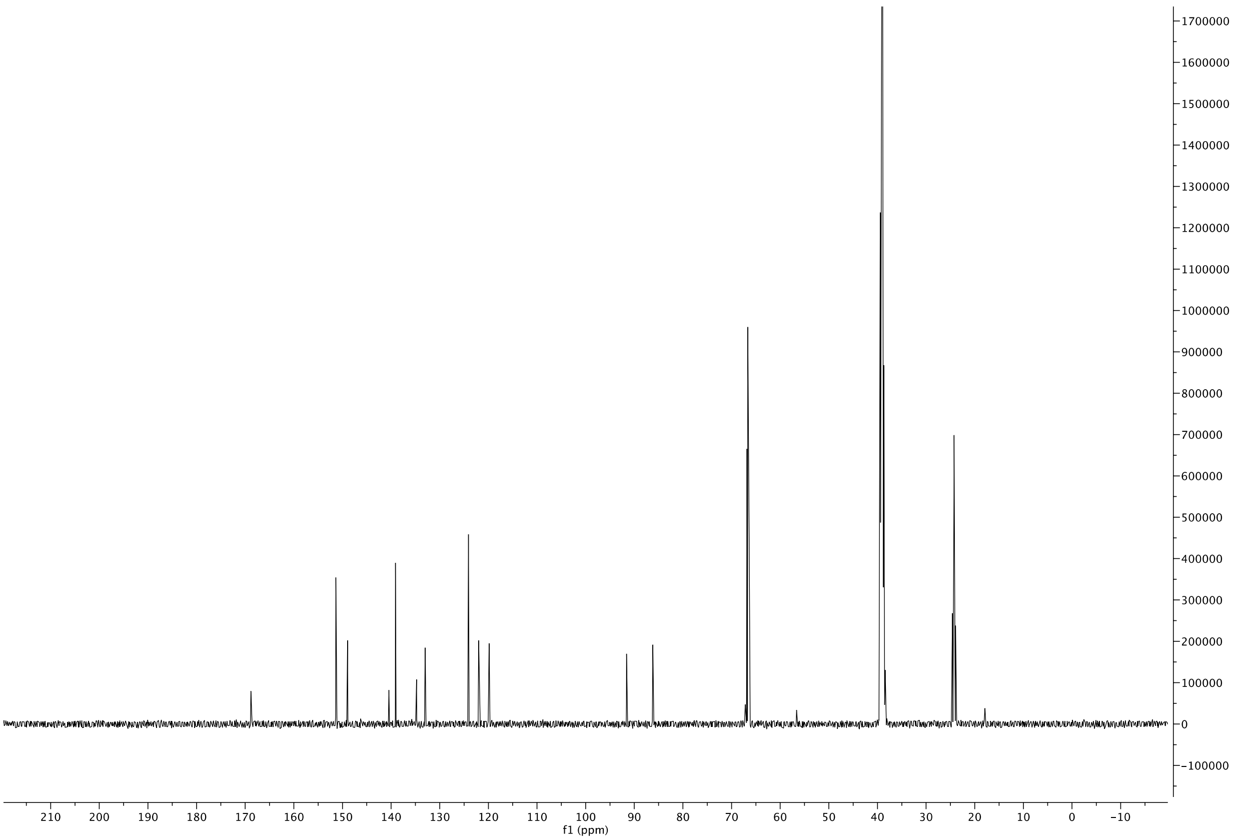

Supplementary Figure S21. $^{13}C\{^{1}H\}$ NMR (151 MHz, 25 °C, DMSO-d_6 / THF-d_8 mixture) spectrum of **L1 Acid**. Residual ethanol resonances are visible at 17.9 and 56.6 ppm.

*Polymer Ligand (**PL**)*

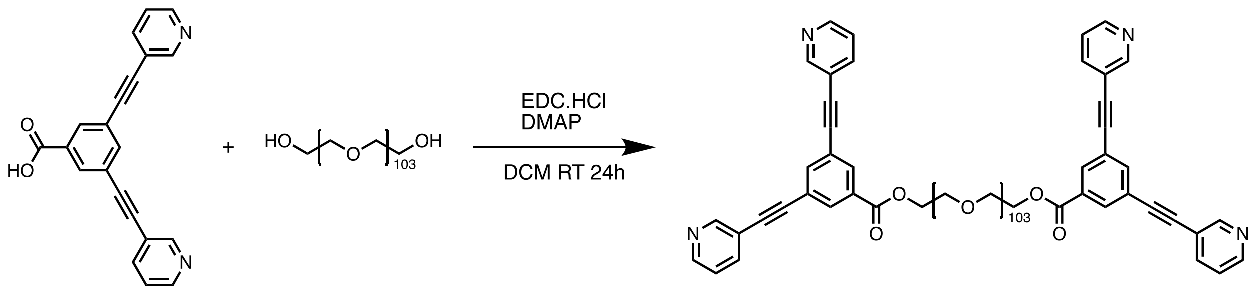

Bis-ligand acid (1.5g, 4.6 mmol, 3 equiv.), poly(ethylene glycol) (Mn average 4600 Da) (7g, 1.53 mmol, 1 equiv.), 1-(3-dimethylaminopropyl)-3-ethylcarbodiimide hydrochloride (EDC.HCl) (1.8g, 9.2 mmol, 6 equiv.), and 4-dimetylaminopyridine (DMAP) (56 mg, 0.46 mmol, 0.3 equiv.) were added to a 100 mL round bottom flask and flushed with nitrogen 3 X. 20 mL of anhydrous DCM was added to the flask and it was allowed to stir at room temperature for 24 hours. The reaction mixture was then diluted with 100 mL of DCM and washed with water (100 mL × 2) and brine (100 mL × 1). The organic phase was dried with Na_2SO_4, filtered, and concentrated to ~ 10mL. The concentrated mixture was added dropwise to rapidly stirring diethyl ether (500 mL) to precipitate the product, which was collected via filtration. This precipitation procedure was repeated twice more, and the product was dried under vacuum at room temperature. 6 g of a white powder was obtained. (76% isolated yield).

^{1}H NMR (500 MHz, $CDCl_3$): δ 8.83 (4H), 8.63 (4H), 8.24 (4H), 7.94 (2H), 7.92 (4H), 7.41 (4H), 4.55 (4H), 3.9-3.5 (460H).

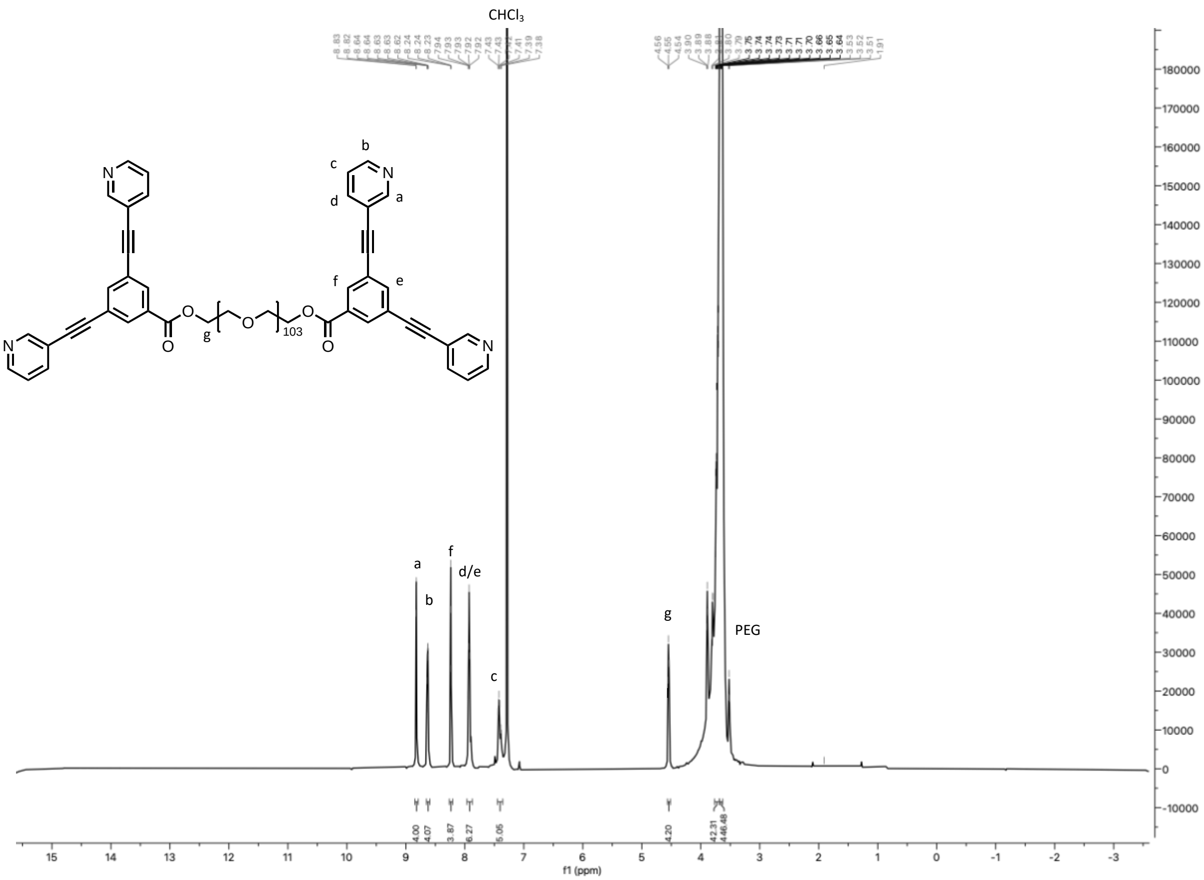

Supplementary Figure S22. ^{1}H NMR (500 MHz, 25 °C, $CDCl_3$) spectrum of **PL**.

L2

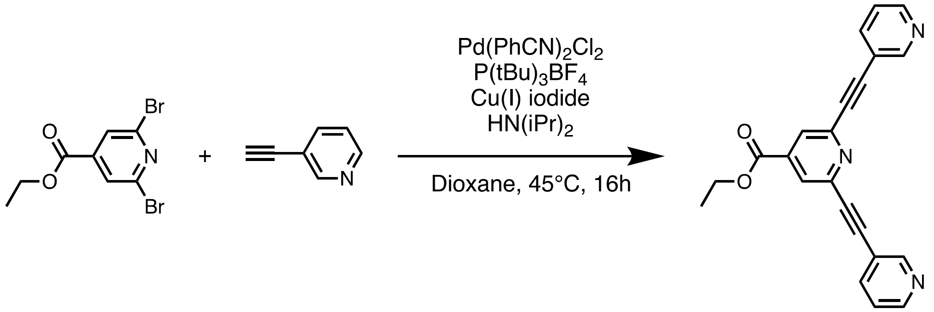

Ethyl-3,5,-dibromoisonicotinate (5g, 16.2 mmol, 1 equiv.), 3-ethyylpyridine (4.3 g, 40.5 mmol, 2.5 equiv.), copper(I) iodide (117 mg, 0.61 mmol, 0.036 equiv), and tri-tert-butylphosphonium tetrafluoroborate (704 mg, 2.43 mmol, 0.15 equiv.) were added to a round bottom flask and which was sealed and flushed with nitrogen 3 times. 200 mL of anhydrous dioxane was added via cannula into the reaction flask, followed by 20 mL of diisopropylamine. The reaction mixture was sparged with nitrogen for 60 minutes before bis(benzonitrile)palladium(II) dichloride (466 mg, 1.215 mmol, .075 equiv.) was added under positive nitrogen pressure. The reaction mixture was further spared with nitrogen for 15 minutes and let stir at 45°C for 16 hours before being cooled to room temperature and filtered through Celite and washed with ethyl acetate until the filtrate was colorless. The resulting dioxane/ethyl acetate solution (~500 mL) was washed with 10% aqueous ethylene diamine (250 mL × 2) and brine (500 mL × 1) before drying the organic layer with Na_2SO_4, filtering, and concentrating to dryness. The crude product was purified using silica column chromatography (0-8% MeOH in DCM gradient, product eluting at 4% MeOH). 3.8 g of a pale-yellow solid was obtained (66% isolated yield). For experiments involving MOC assembly in solution, the product was further purified via recrystallization from boiling toluene.

^{1}H NMR (500 MHz, $CDCl_3$): δ 8.86 (dd, *J* = 2.1, 0.9 Hz, 2H), 8.63 (dd, *J* = 5.0, 1.7 Hz, 2H), 8.07 (s, 2H), 7.95 (dt, *J* = 7.9, 1.9 Hz, 2H), 7.38 (ddd, *J* = 7.9, 5.0, 1.0 Hz, 2H), 4.47 (q, *J* = 7.1 Hz, 2H), 1.45 (t, *J* = 7.1 Hz, 3H).

^{13}C{^{1}H} NMR (151 MHz, $CDCl_3$): δ 163.73, 152.11, 149.04, 144.12, 139.65, 138.99, 126.06, 123.43, 119.29, 90.93, 86.95, 62.46, 14.23.

DART-HRMS (m/z) calculated for ($C_{22}H_{15}N_3O_2$) = 354.1264 $[M+H]^+$; found 354.1283

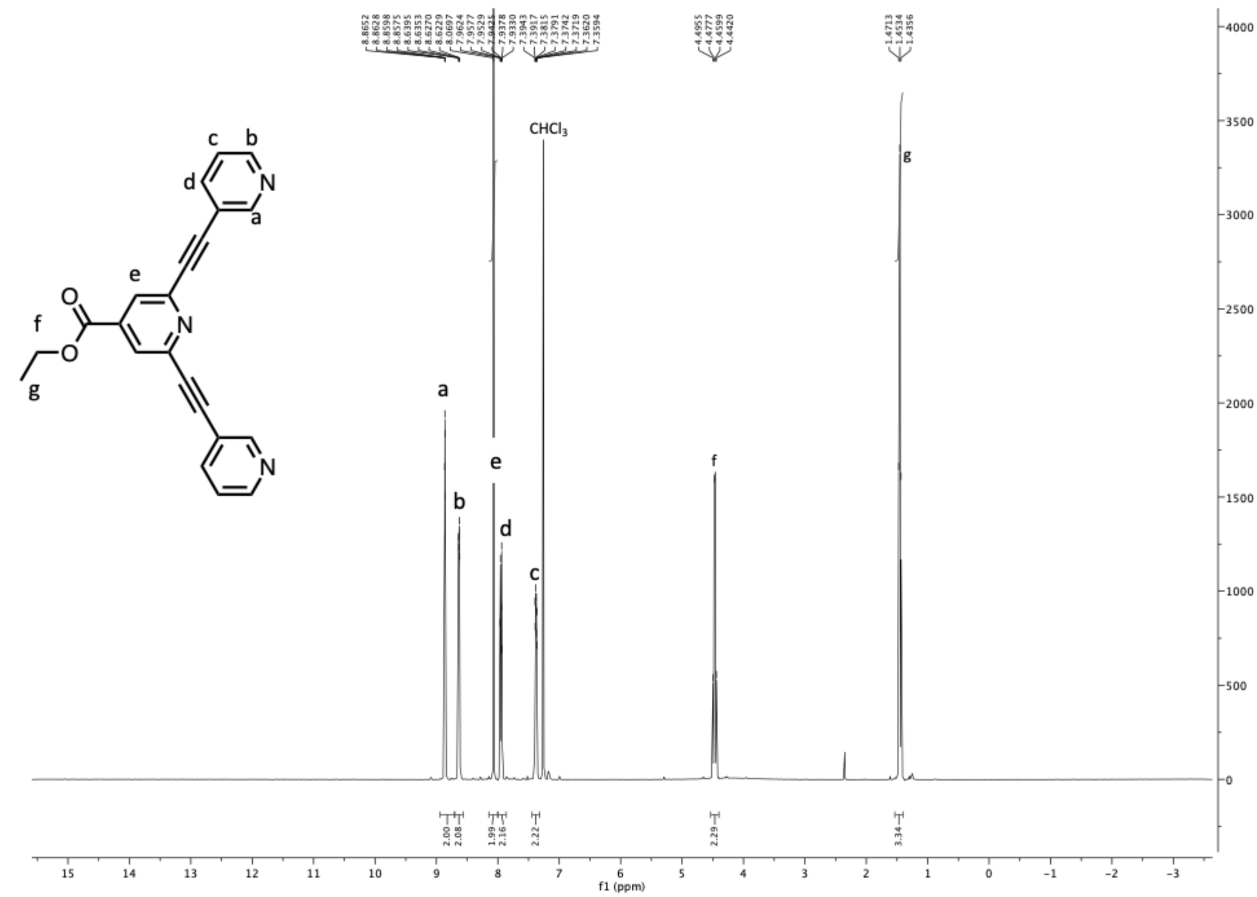

Supplementary Figure S23. ^{1}H NMR (500 MHz, 25 °C, $CDCl_3$) spectrum of **L2**.

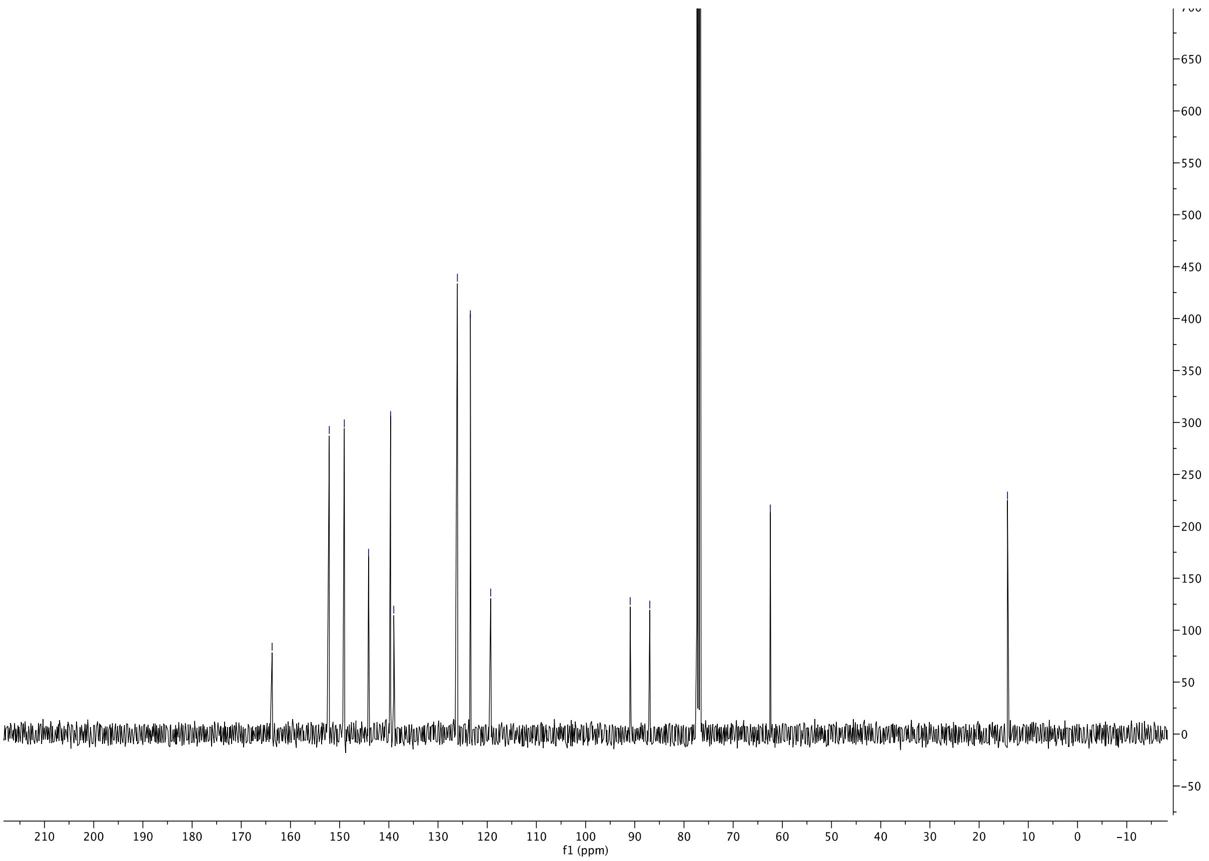

Supplementary Figure S24. $^{13}C\{^{1}H\}$ NMR (151 MHz, 25 °C, $CDCl_3$) spectrum of **L2**.

MOC Synthesis - With Added Guest

For the synthesis of MOCs containing added guest (for guest binding studies, crystallography, DOSY characterization, etc.) a slightly modified procedure was used as the '*No Added Guest*' case. Here, more concentrated DMSO solutions of **L1** or **L2** and $Pd(MeCN)_4(OTf)_2$ were prepared and first mixed together to form a cursory solution of **MOC** or **N-MOC**. Typically, the total volume of these solutions was ~100-200 μL. We note that $Pd(MeCN)_4(OTf)_2$ is readily soluble in DMSO however **L1** or **L2** solutions with concentrations >~1mg/50 μL were briefly heated to speed up dissolution. To this mixed solution was added the guests from concentrated stock solutions of each guest in DMSO (the concentration varying depending on the intended application).

MOC Synthesis - With Excess Palladium

MOC assemblies with excess palladium were synthesized identically to the '*No Added Guest*' case however a more concentrated $Pd(MeCN)_4(OTf)_2$ was created to allow for higher Pd^{2+} equivalents while maintaining a final solution volume of 500 μL. In Supplementary Figure S31, the 'equivalents' label in the legend denotes a multiple of the standard amount of Pd^{2+} used for (poly)MOC assembly.

PolyMOC Fabrication with Added Guest

PolyMOC gels with included guest were fabricated in an analogous procedure to those without, but in these cases a solution of guest in DMSO was added to the stock solution of **PL** before the introduction of palladium (the overall concentration of polymer ligand was constant in all cases). For all guests sufficient equivalents were added to achieve >99% of cage binding occupancy based on the previously measured association constants. These equivalents are listed in Supplementary Table S1.

Supplementary Table S1. Measured Association Constants for guests with **MOC** and the corresponding equivalents of guest added for polyMOC gel fabrication.

Guest	Association Constant (M^{-1})	Equivalents of Guest Added
**HSO_4**	1,000 ± 500	4.9
**NO_3**	6,000 ± 1,000	1.5
**DAQ**	62,000 ± 2,000	1.1

Excess Palladium polyMOC Gel Synthesis

PolyMOC gels with excess palladium were fabricated analogously to those on-stoichiometry but using a more concentrated stock solution of $Pd(MeCN)_4(Otf)_2$ unless otherwise noted. Compositions containing sufficient equivalents of palladium to preclude gelation were simply mixed on a vortex to homogenize after addition of palladium. During our studies we observed some mixtures turning opaque dark brown/black upon prolonged annealing, possibly due to

palladium reduction. To prevent this, these materials were subjected to a modified annealing procedure (15 minutes at 50°C).

Additional Small-molecule Ligand polyMOC Gel Synthesis

PolyMOC gels containing a mixture of **PL1** and **L2** were fabricated analogously to the '*General Fabrication Procedure*' however slightly more concentrated stock solutions of PL1 were used to allow for the addition of a controlled amount of **L2** from an additional stock solution. These gels were cast and annealed analogously.

^{1}H NMR CP-MAS Characterization of PolyMOC Gels

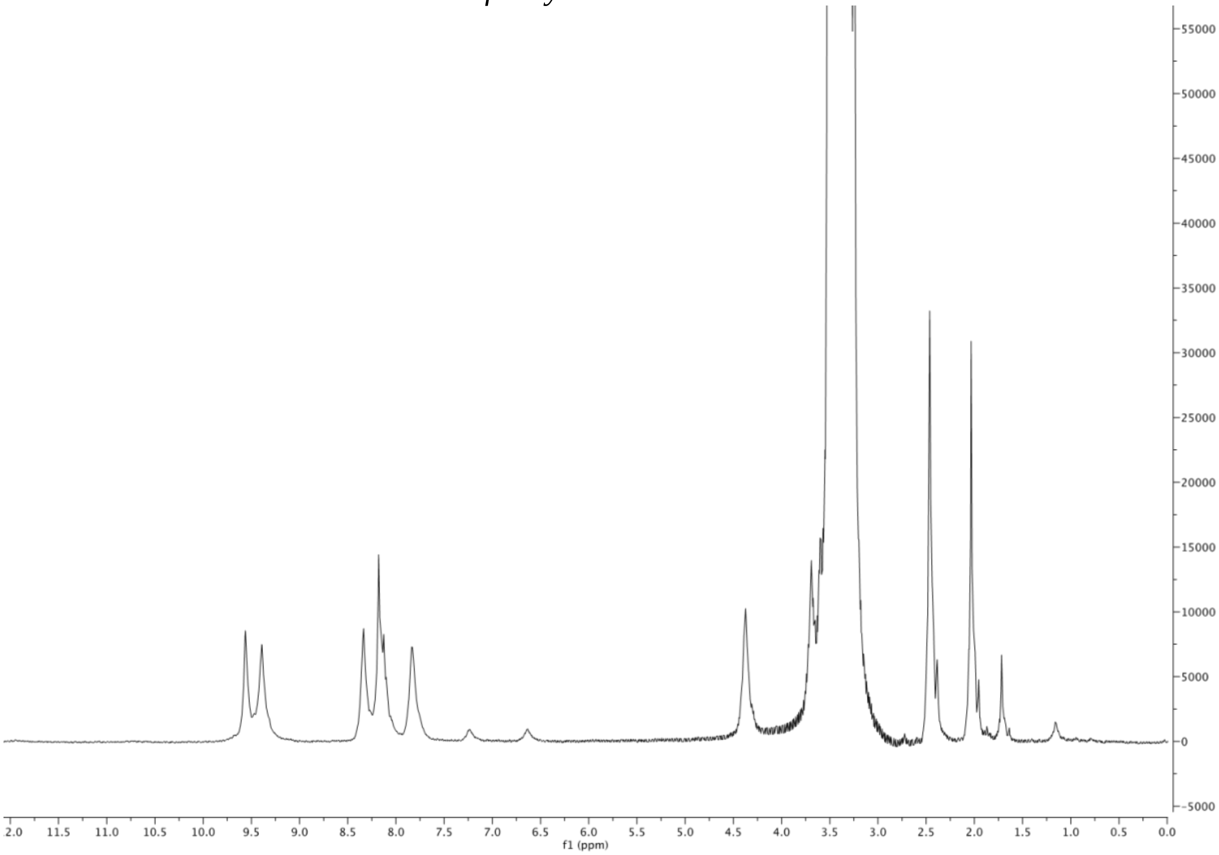

Fig. S25. ^{1}H CP-MAS NMR spectrum of **poly(OTf⊂MOC)** (500 MHz, 25 °C, DMSO-d_6).

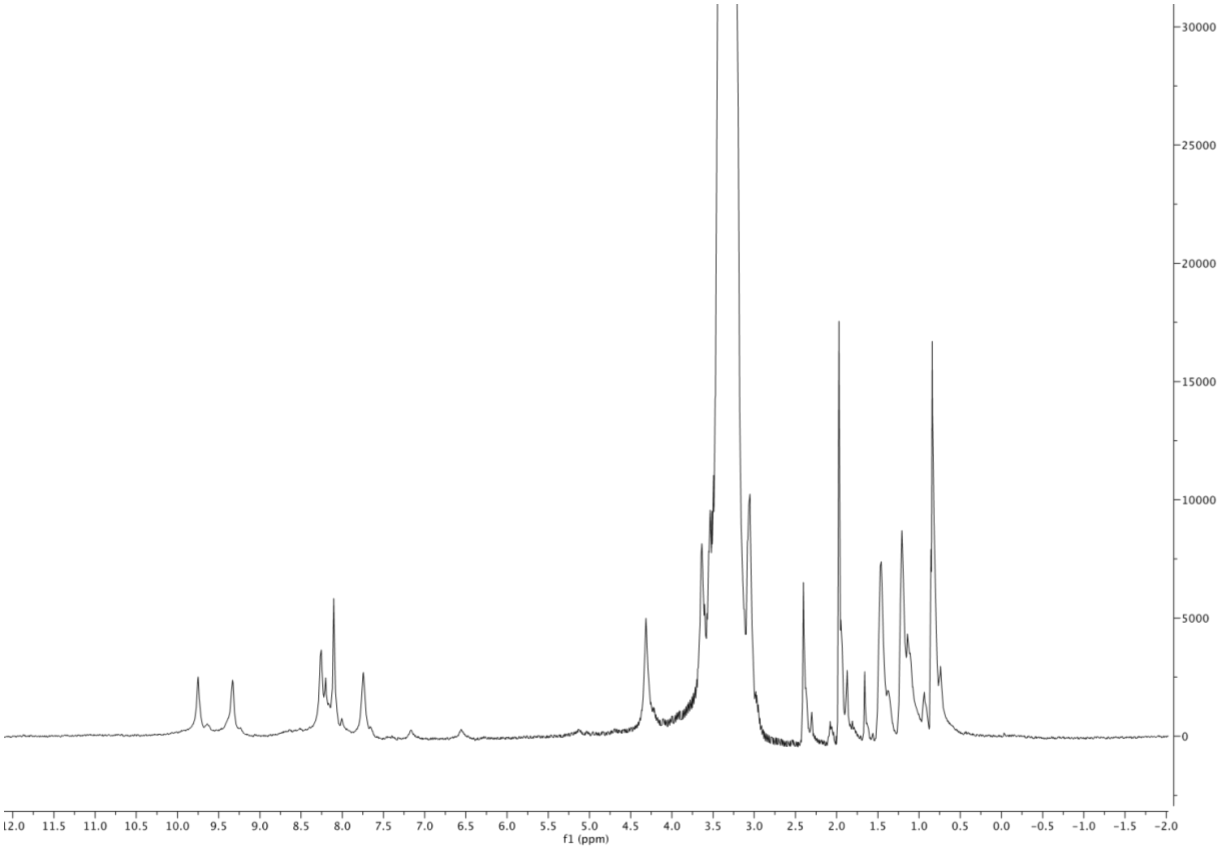

Fig. S26. ^{1}H CP-MAS NMR spectrum of **poly(HSO_4⊂MOC)** (500 MHz, 25 °C, DMSO-d_6).

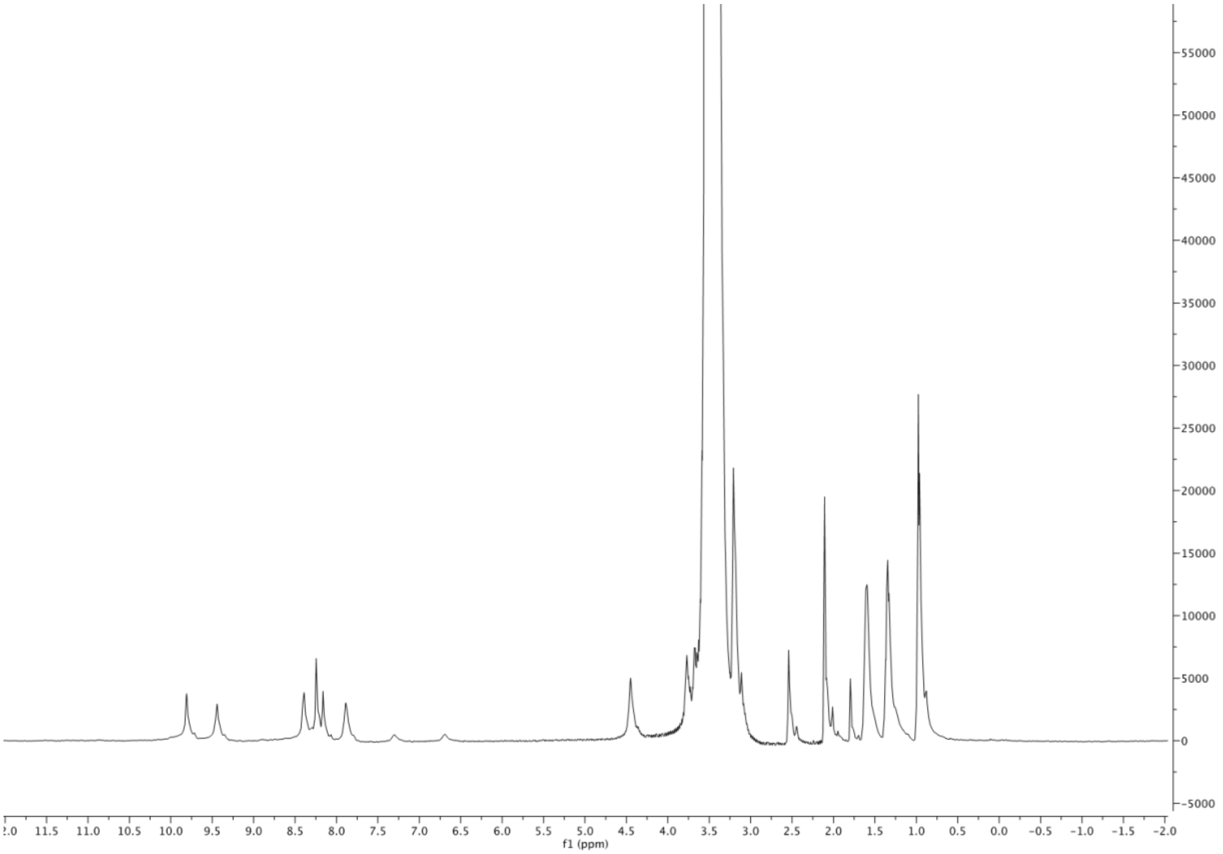

Fig. S27. ^{1}H CP-MAS NMR spectrum of **poly(NO_3⊂MOC)** (500 MHz, 25 °C, DMSO-d_6).

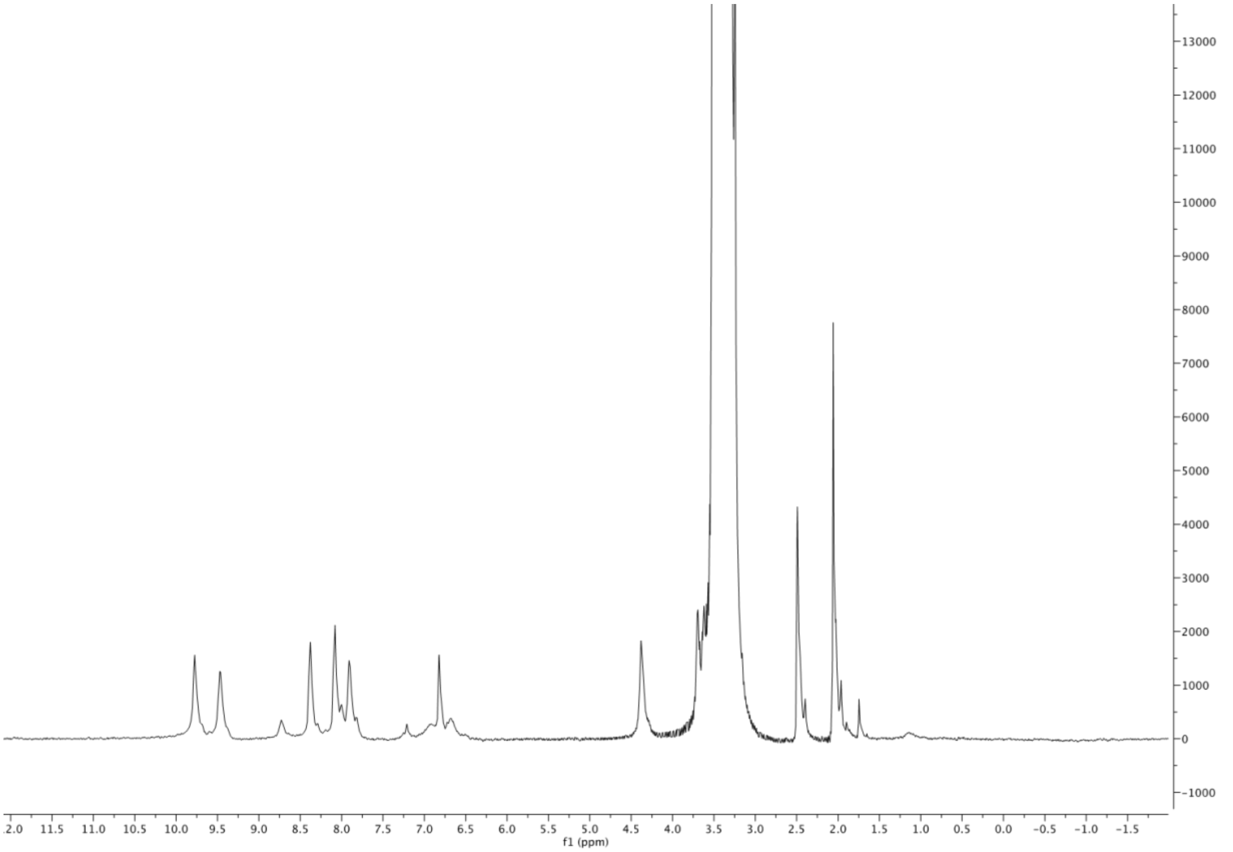

Fig. S28. ^{1}H CP-MAS NMR spectrum of **poly(DAQ⊂MOC)** (500 MHz, 25 °C, DMSO-d_6).

Representative Frequency Sweep Data for PolyMOC Gels

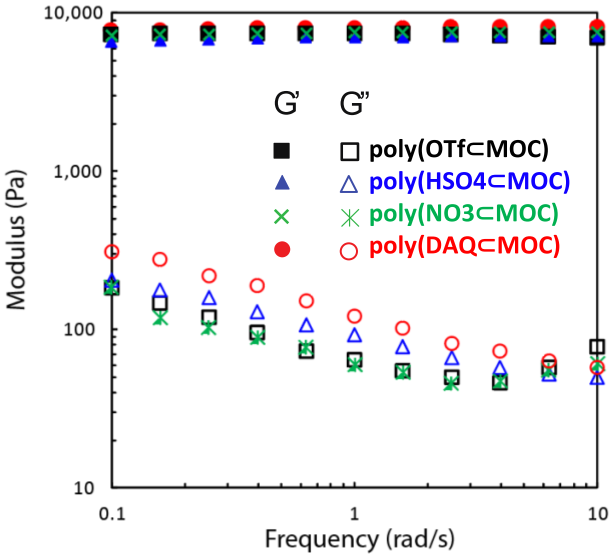

Supplementary Figure S29. Representative frequency sweeps for polyMOC gels fabricated according to the general procedure. Data were acquired using an 8mm geometry with gap sizes ~ 1mm at room temperature.

Stress Relaxation Modeling

The stress relaxation experiments for all gels were fit to the stretched exponential equation:

$$G(t) = G_0 \exp\left(-\left(\frac{t}{\tau}\right)^{\beta} \right)$$

The stretched exponential can be characterized as a distribution of relaxation times, however the specific value of τ fit in the above expression does not correspond to a specific value of this distribution (i.e. the mean, median, or higher order moments), so instead calculate the mean relaxation time of this distribution as follows:

$$\langle\tau\rangle = \frac{\tau\Gamma(1/\beta)}{\beta}$$

Where Γ is the gamma function.

Modeling of Excess Pd^{2+} and Mixed-Ligand PolyMOC Network Structures

To design the multi-state switching material illustrated in Figure 6 of the main text, we used modeling based on classical network formation theories (e.g., Miller-Macosko theory) to make cursory predictions on the mechanical properties of polyMOC gels derived from mixtures of polymer ligand and small-molecule ligand. An ideally formed polyMOC gel with a bifunctional polymer ligand which forms Pd_2L_4 structures can be viewed as an A_2 + B_4 type network structure with a 2:1 stoichiometry (which implies an equimolar amount of A and B functionalities in the system; following the nomenclature of Miller and Macosko, the stoichiometric ratio r = 1). At complete conversion (i.e., when all A and B groups have reacted with each other) this system forms a percolated network as observed experimentally and as predicted by theory (*vide infra*). We can view both the super stoichiometric Pd^{2+} system and the small-molecule ligand plus polymer ligand system as either decreasing the *proportion* of the B component to the A component (r < 1) or as decreasing the *conversion* of all functional groups (e.g., extent of reaction p is <1), however each case is more intuitively described by a single method. In general, for an A_2 + B_f system, gelation is predicted to occur at a critical extent of reaction, p_c given by the following equation:

$$p_c = \frac{1}{\sqrt{r(f_w - 1)}} \quad \text{eq. 1}$$

Where r is the stoichiometric ratio and f_w is the weight-averaged branching functionality of the B component. For r = 1, f_w = 4, this value is ≈ 0.578.

When super stoichiometric Pd^{2+} is added to the system, we observed the somewhat clean mixture of MOC and non-cage forming ligand (presumed to be coordinating to two palladium ions each), therefore this system can be thought of as decreasing the stoichiometric ratio of B_4 component to A_2 component. The relationship between the stoichiometric ratio, r, and the fraction of bis-pyridine ligands forming MOC structures, ϕ_{MOC}, is:

$$r = 1 - \phi_{MOC} \qquad \text{eq. 2}$$

Plugging Eqn. S2 into Eqn. S1, we get:

$$p_c = \frac{1}{\sqrt{(1-\phi_{MOC})(f_w - 1)}} \qquad \text{eq. 3}$$

For our system we consider the extent of reaction to be equal to 1, which allows us to calculate the critical fraction of ligands which must exist in MOC structures to maintain gelation, $\phi_{MOC,critical}$, as:

$$p_c = 1 = \frac{1}{\sqrt{(1-\phi_{MOC})(4-1)}} \Longrightarrow \phi_{MOC,critical} = 2/3 \qquad \text{eq. 4}$$

In real systems, topological defects will require the fraction of ligands forming MOC structures to be larger than the value calculated above.

For systems constructed from a mixture of polymer bound ligands and small-molecule ligands in a super stoichiometric ratio to added Pd^{2+}, the same number of MOCs will always be formed, however the connectivity of each ligand will be changed as a guest is added to drive self sorting. We consider a general case where the ratio of small-molecule ligands to polymer bound ligands is equal to $\rho_{SML:PL}$ and the ratio of total bispyridine ligands to palladium ions is $\rho_{L:Pd^{2+}}$. Based on these ratios, the stoichiometric ratio, *r*, between A_2 polymer bound ligands and B_4 MOC structures is:

$$r = \frac{B_4 MOC\ Structures}{A_2 Polymer\ Bound\ Ligands} = \frac{1+\rho_{SML:PL}}{\rho_{L:Pd^{2+}}/2} \qquad \text{eq. 5}$$

We note that the value of *r* is <1 for further analysis. We can view the preferential incorporation of polymer bound ligand into MOC structures as a selective guest is added to be equivalent to the increasing the extent of reaction, *p*, of the A_2 functionalities. Initially, if there is no preference for self-sorting, the fraction of polymer bound ligands incorporated into MOC structures is equal to:

$$\gamma_{MOC} = \left(\frac{1}{\rho_{SML:PL}+1}\right)\frac{1}{2\rho_{SML:PL}} \quad \text{eq. 6}$$

Here we define a preferential self-sorting factor, ϕ, which is equal to the preferential fraction of polymer-bound ligands incorporated into MOC structure. This factor serves to multiple the value of γ_{MOC} in our analysis and can be observed empirically as was shown in Figure 6B. We note that the ratio $\rho_{SML:PL}$ is of the individual ligand moieties, and thus a single molecule of **PL** contains *two* polymer bound ligands. γ_{MOC} is equal to the extent of reaction, *p*, for this system as well. To form a gel, this value needs to be above the critical value defined as:

$$\gamma_{MOC} = \left(\frac{1}{\rho_{SML:PL}+1}\right)\frac{\phi}{2\rho_{SML:PL}} > p_c = \frac{1}{\sqrt{\frac{1+\rho_{SML:PL}}{\rho_{L:Pd^{2+}}/2}(f_w-1)}} \qquad \text{eq. 7}$$

The above equation is what guided our development of the modified ratio of **L2** : **PL** : $\mathbf{Pd^{2+}}$ used for the guest-triggered sol-gel transitions driven by (reversible) self-sorting. We note that the 1:1 mixture of **L2** and **L1** used for the studies in Figure 6B/6C in a 33% excess to Pd^{2+}, when translated into a polyMOC gel (i.e., replacing all **L1** with half an equivalent of **PL**) produced a free-flowing material that did not gel upon extended annealing. This result was initially rationalized intuitively, as an equimolar ratio of small molecule and polymer-bound ligands with no preference for self-sorting would produce on average MOC network junctions with a number-average branching functionality of 2, with a lower than quantitative incorporation of polymer bound ligand into MOC structures. Further, a quantitative rationalization of this observation is provided by analysis using eq. S7.

Practically, we sought an approach that would reduce the predictive complexity of eq. S7. Therefore, we began with the standard **poly(OTf⊂MOC)** recipe to which excess equivalents of **L2** were added until the material no longer resulted in a freestanding gel. This approach ensured that *r* was equal to 1, and only the fraction of polymer-bound ligands attached to MOC structures was modulated as **L2** was introduced. For this case, we plot the critical and predicted extents of reactions as a function of equivalents of L2 added in Supplementary Figure S30 below.

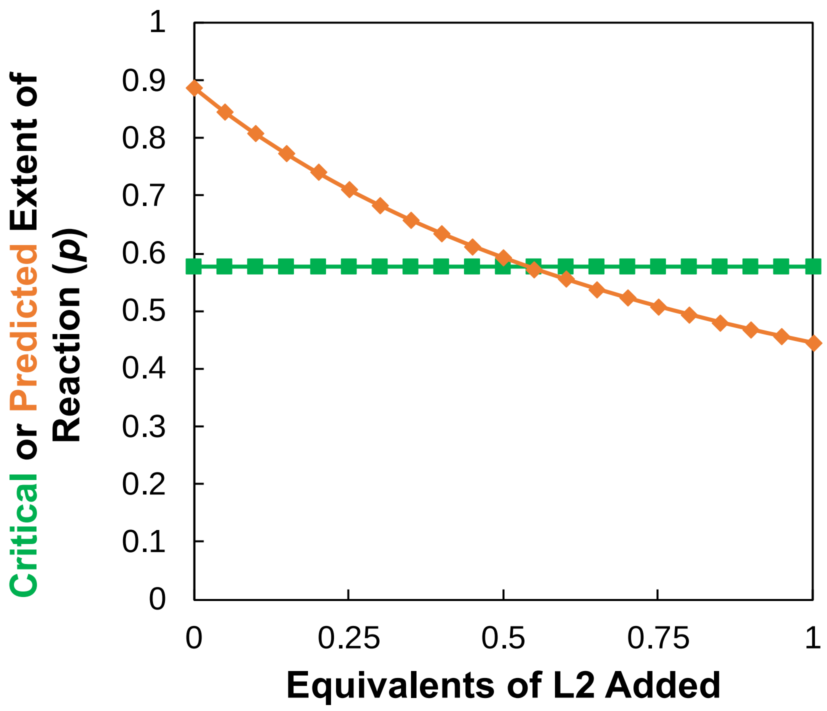

Supplementary Figure S30. Critical or Predicted extent of reaction as a function of excess equivalents of **L2** added. For this case, the ratio of PL : Pd^{2+} is held constant at 1 : 1 (an initial on-stoichiometry mixture) and no preferential ligand self-sorting is considered.

As shown in Supplementary Figure S30, gelation is predicted if the predicted extent of reaction is above the critical extent. According to theory, the transition from sol to gel should occur when > ~0.5 equiv. **L2** are added, however experimentally we observe this transition around ~0.25 equiv. **L2**. The direction of this deviation is expected based on the assumptions present in the classical network formation theories. By not considering real network topology, we *overestimate* the predicted extent of reaction by ignoring the possibility for elastically ineffective network defects.

Characterization of Gels Fabricated with Excess Pd^{2+}

MOC and polyMOC assembly was further characterized under assembly conditions containing super stoichiometric Pd^{2+}. MOCs were assembled under conditions with increasing equivalents of Pd^{2+} (relative to the standard conditions, 2 bispyridine ligands per palladium ion) and the fraction of assembled cages was determined by integration of the resonances of proton Ha of the MOC and unbound ligand, as illustrated in Figure 6B. Supplementary Figure S31A shows the fraction of MOCs assembled as a function of excess Pd^{2+} with and without the inclusion of 1 equiv **DAQ**. Inclusion of **DAQ** serves to stabilize MOC formation and leads to higher fractions of MOC assembly under the same amount of super stoichiometric Pd^{2+}. Further, the fraction of cage assembly is proportional to guest equivalents, as a sample containing 0.5 equiv **DAQ** saw an intermediate fraction of MOC formation between 0 and 1 equiv. An analogous experiment was conducted in the polyMOC system, for which the storage moduli of the materials as measured at 1 rad/s is reported in Supplementary Figure S31B. Stress relaxation studies of a material containing 3 equiv Pd^{2+} were conducted and the results are shown in Supplementary Figure S31C. we find that gels containing 3 equiv. Pd^{2+} display a shorter relaxation time than on-stoichiometry assembly, likely due to their lower modulus and higher fraction of unbound ligands, resulting in lower cross-linker functionality networks, which are known to have display shorter relaxation times than higher functionality networks. Addition of guest under these super stoichiometric Pd^{2+} conditions lead to an increase in relaxation time and a concomitant increase in modulus (as shown in Supplementary Figure S31B), however MOC assembly is not completely recovered and thus the relaxation time is still moderately shorter than than on-stoichiometry polyMOC containing DAQ. CP-MAS 1H NMR spectra of a polyMOC assembled under super stoichiometric Pd2+ conditions (3 equiv.) with and without 1 equiv. added **DAQ** is shown in Figures S32-S33. When no guest is added, some disassembly is observed, analogous to our observations in small-molecule MOC studies. Upon addition of 1 equiv. **DAQ**, distinct downfield shifts of cage resonances are observed, mirroring our observations in well-assembled **poly(OTf⊂MOC)**, indicating that guest binding occurs under super stoichiometric Pd^{2+} conditions. However, complete poly(MOC) assembly is not observed in this case, consistent with measurements of MOC assembly and storage modulus shown in Supplementary Figure S31.

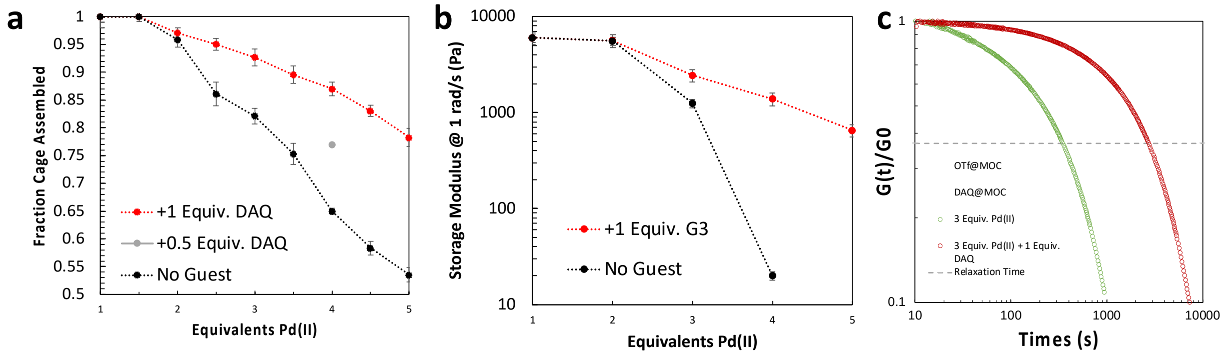

Supplementary Figure S31. Characterization of (poly)MOC systems with varying equivalents of Pd^{2+}. **A.** MOC assembly with varying equivalents of Pd^{2+} (1 to 5 equiv.) with and without 1 equiv. **DAQ** as determined by integration of resonances associated with H_a of **L1-Pd₂** and **DAQ⊂MOC** (see Figure 6B) **B.** Storage moduli (G') of polyMOC gels assembled with varying equivalents of Pd^{2+} with and without 1 equiv. **DAQ**. **C.** Normalized stress relaxation data for polyMOC gels containing 3 equiv. Pd2+ with and without 1 equiv. DAQ. Data are plotted alongside those for **poly(DAQ⊂MOC)** and **poly(OTf⊂MOC)** for reference (see Figure 5A).

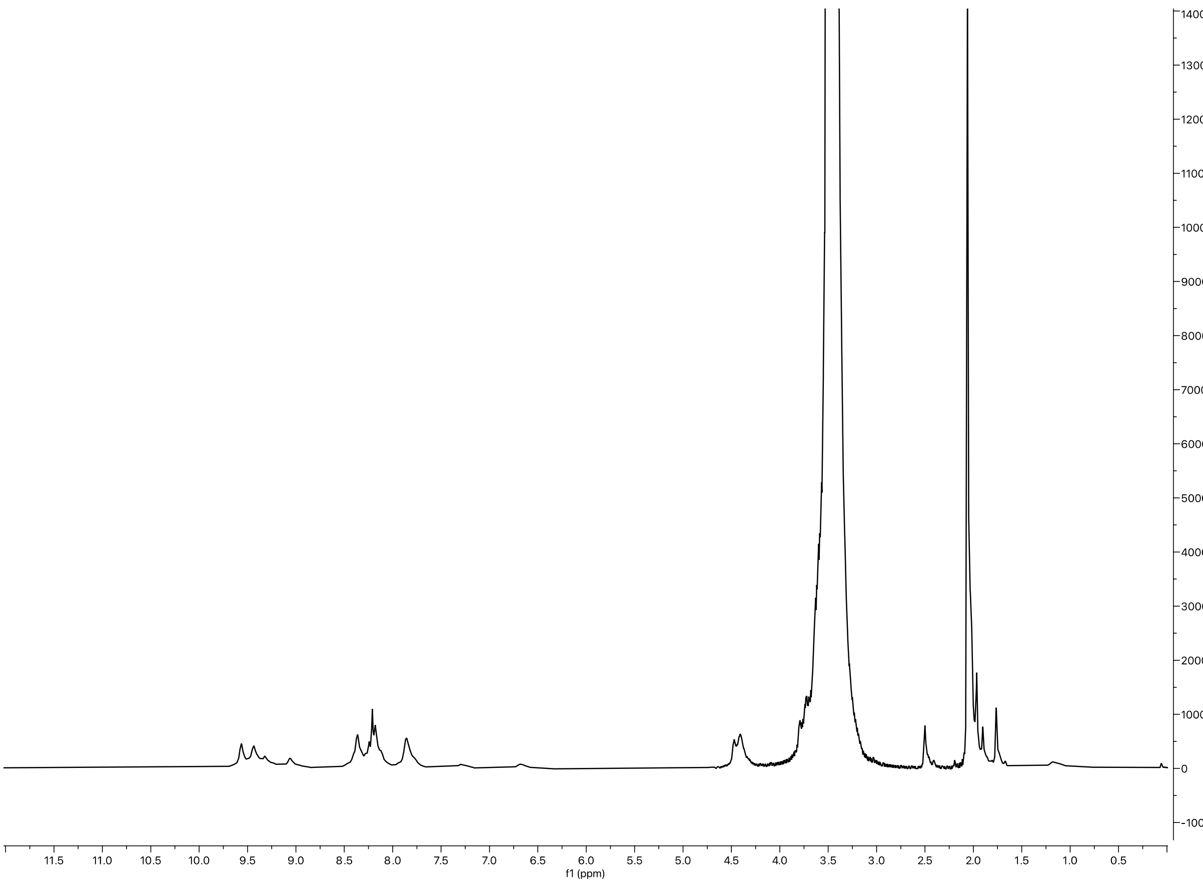

Supplementary Figure S32. ^{1}H CP-MAS NMR spectrum of **poly(OTf⊂MOC)** assembled with 3 equiv. Pd^{2+} (500 MHz, 25 °C, DMSO-d_6).

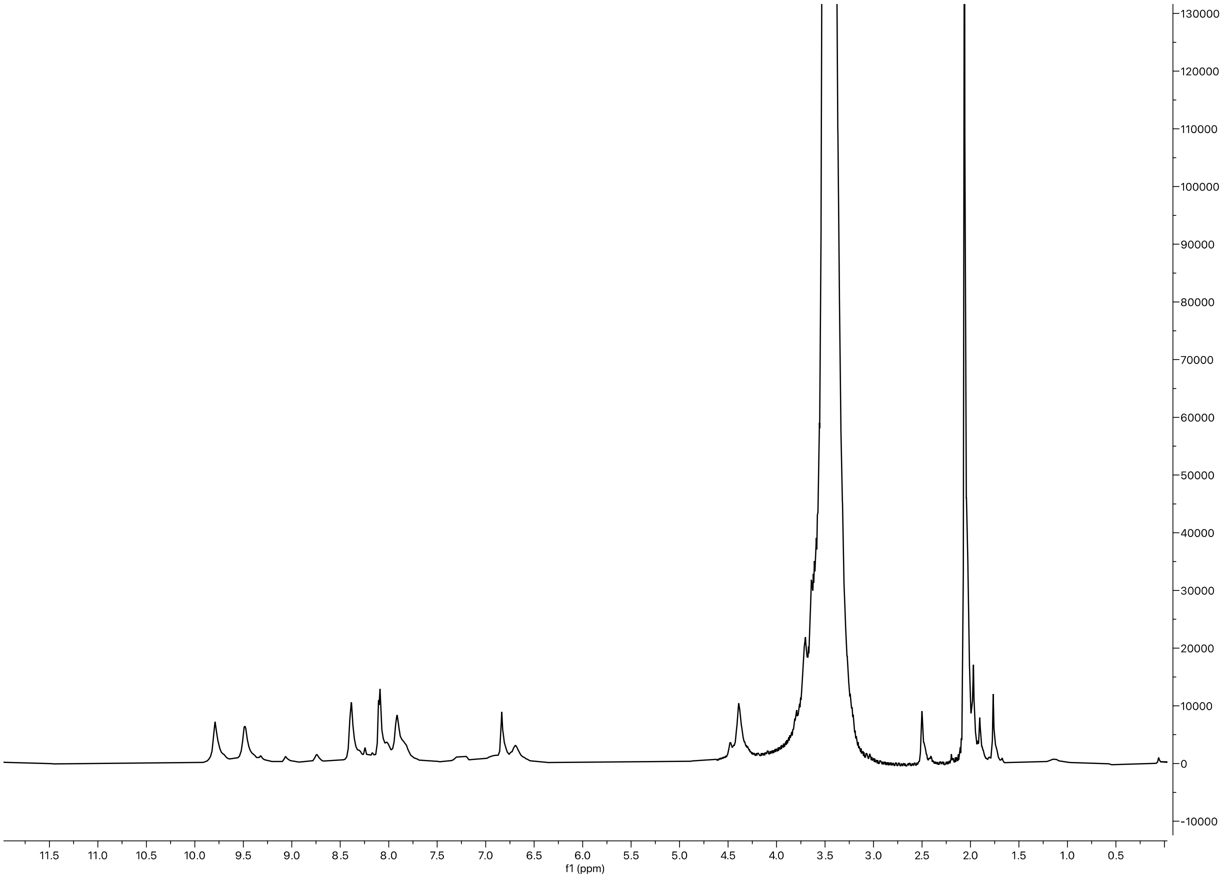

Supplementary Figure S33. ^{1}H CP-MAS NMR spectrum of **poly(OTf⊂MOC)** assembled with 3 equiv. Pd^{2+} and 1 equiv. **DAQ** (500MHz, 25 °C, DMSO-d_6).

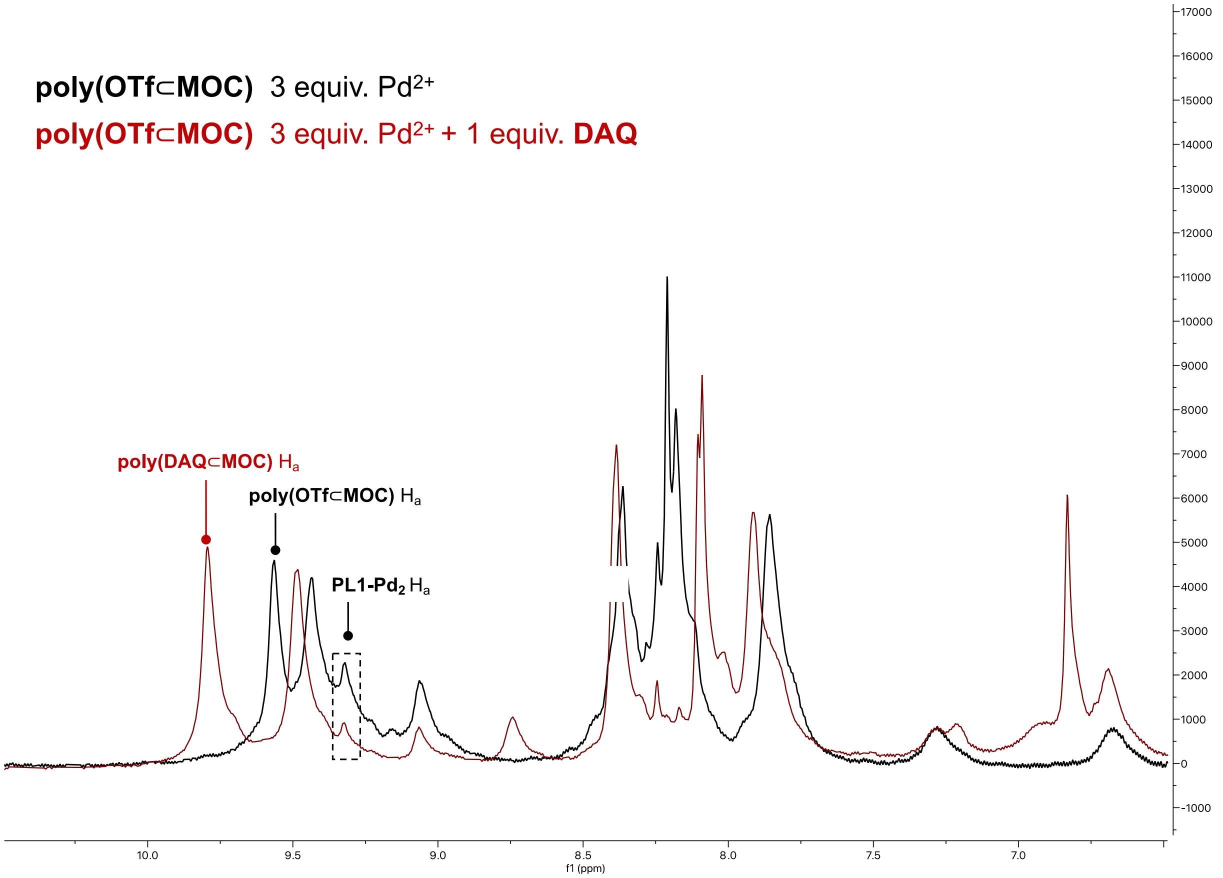

Supplementary Figure S34. Detail of the spectra shown in Figures S32-S33 superimposed. Resonances of the **DAQ**-bound MOC junction (H_a) are distinctly shifted down-field compared to the sample with no **DAQ**, indicative of guest binding. Further, resonances of PL1 moieties bound to 2 Pd^{2+} ions are observed similarly to small-molecule MOC studies (see Figure 6B).

Guest-Triggered Sol-Gel Transitions

For these studies (depicted in Figure 6D) an initial mixture containing **PL1**, **L2**, and **Pd^{2+}** was fabricated according to the '*Additional Small-molecule Ligand polyMOC Gel Synthesis*' procedure listed above. This mixture contained a **PL1** : **L2** : **Pd^{2+}** ratio of 1 : 0.25 : 1 (0.01 mmol, 0.0025 mmol, and 0.01 mmol of each component, respectively, in 500 uL of DMSO). This proportion was fabricated on a 3 mL scale to allow for sol-gel transitions to occur in the 'mother' mixture from which pieces could be removed, massed, and characterized by rheometry. We found that this method, as opposed to trying to realize multi-state switching behavior in a single gel puck greatly expedited mechanical characterization and reproducibility. Initially our mixture was a free-flowing liquid, however upon addition of 0.8 equiv. **DAQ** from a concentrated stock solution (100 mg/mL) and subsequent annealing (15 minutes at 50°C, the standing annealing conditions used in this procedure), the mixture became a freestanding gel. This gel was homogenized within the vial using a spatula and a portion was removed, carefully massed, and cast into a puck for rheometry. To the mother vial was subsequently added 3 equiv. **NO$_3$** from a concentrated stock solution (100 mg/mL) which was mixed into the material using a spatula and annealed. After annealing the gel was observed to transition into a free-flowing viscous liquid. Using a pipette, a small portion of this liquid was removed, carefully massed, and characterized by rheometry. Subsequently, this procedure was repeated twice more, adding 1 equiv. **DAQ**, 5 equiv. **NO$_3$**, 3 equiv. **DAQ**, and 10 equiv. **NO$_3$**. Representative stress-relaxation experiments for each of the gelled self-sorting systems as shown in Figure 7E was conducted and the results are shown in Supplementary Figure S35. Direct comparison to other polyMOC systems in this work is complicated by the fact that self-sorting systems contained an appreciable fraction of **L2** and were assembled with sub-stoichiometric Pd^{2+}, thus they displayed markedly lower moduli and relaxation times, even in the presence of **DAQ**.

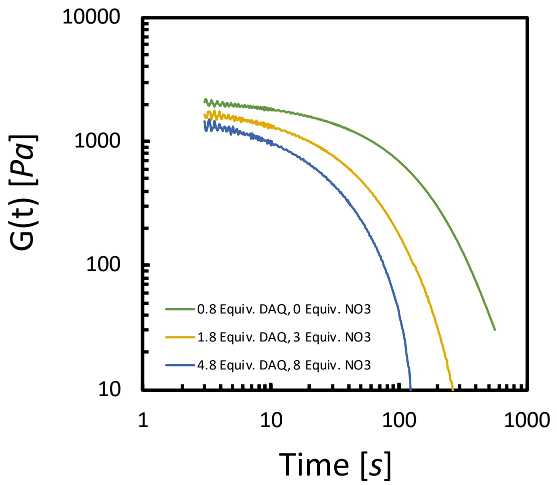

Supplementary Figure S35. Representative stress relaxation studies of the polyMOC gels from the self-sorting studies shown in Figure 7E. A concomitant decrease in relaxation time with modulus after self-sorting is observed, consistent with the lowering of cross-linker functionality due to less-selective MOC formation, similarly to what was observed in stress relaxation studies of polyMOCs assembled under

Guest Uptake into Preformed PolyMOC Gels

For these experiments, polyMOC gels were fabricated using the general procedure from the *PolyMOC Fabrication and Characterization* section of the Supporting Information. 1mL pucks of **poly(OTf⊂MOC)** (containing approximately 0.01 mmol of MOC junction) were placed into ~5mL of 10 mM solutions of guest (**DAQ**) in DMSO and allowed to sit for three hours at room temperature. Over this time, the gel pucks were observed to flow slightly and lose their form but remained as mechanically robust materials. This observation is consistent with the measured relaxation time of **poly(OTf⊂MOC)**. After three hours, the gels were removed from the guest solutions, washed briefly five times with DMSO, and packed into rotors for characterization via CP-MAS ^{1}H NMR (which are shown below). These spectra are shown below and contain resonance shifts in-line with those of the pre-assembled polyMOC gels fabricated in the presence of guest, indicating that guest binding does not require the elevated temperature and increase ligand exchange dynamics present during fabrication to enable guest binding.

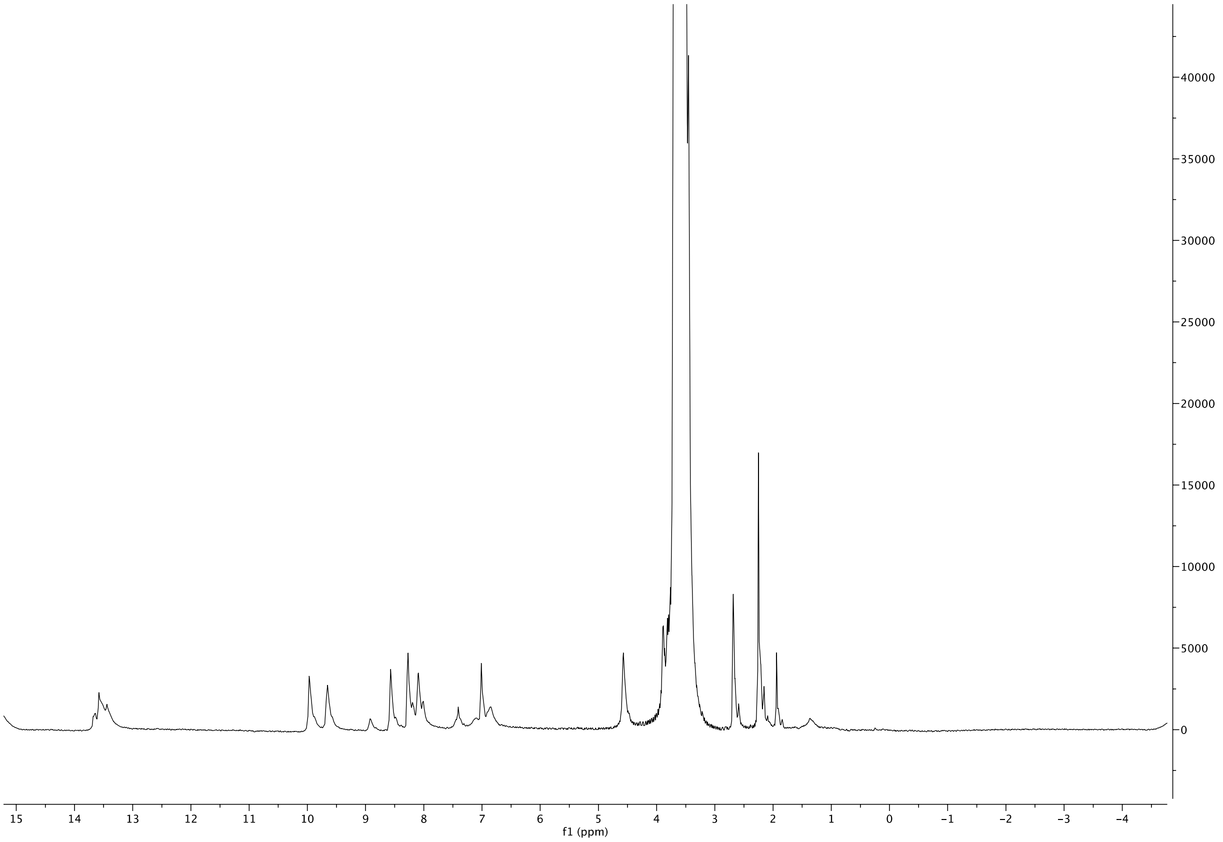

Supplementary Figure S36. ^{1}H CP-MAS NMR spectrum of **poly(OTf⊂MOC)** after sitting in a solution of **DAQ** (500 MHz, 25 °C, DMSO-d_6).

High-Resolution Mass-Spectrometry Characterization of MOCs

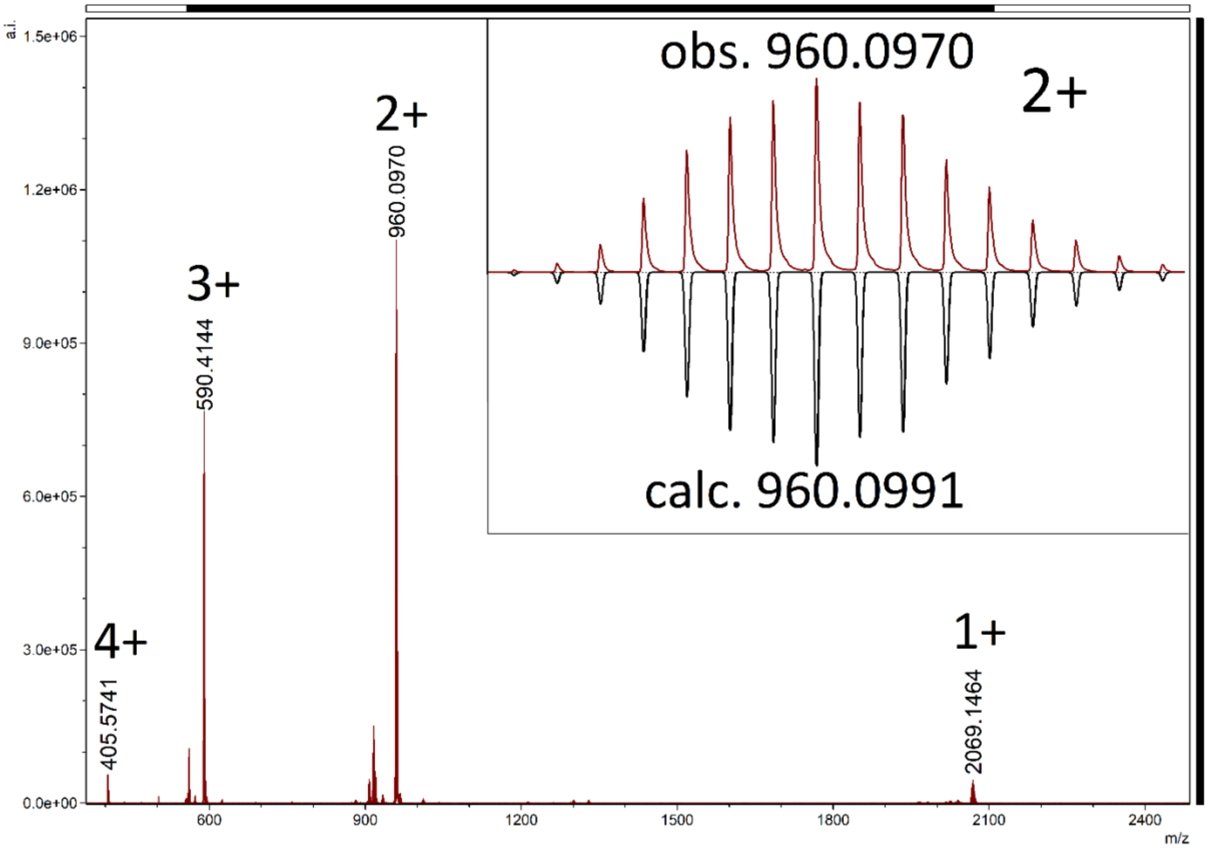

Supplementary Figure S37. Full HR-ESI-MS spectra of **MOC** with annotation to different charged states of $[Pd_2L_4]^{n+}[OTf^-]_{4-n}$ for n= 1-4. Upper right: zoom in into 2+ species showing the measured value and isotopic pattern (top) and the calculated spectra (bottom).

ESI-HRMS (m/z) calculated for $[(Pd_2L_4)(OTf)_3]^{1+}$ = 2069.1509; found 2069.1464. $[(Pd_2L_4)(OTf)_2]^{2+}$ = 960.0991; found 960.0970. $[(Pd_2L_4)(OTf)_1]^{3+}$ = 590.4154; found 590.4144. $[(Pd_2L_4)(OTf)_0]^{4+}$ = 405.5733; found 405.5741.

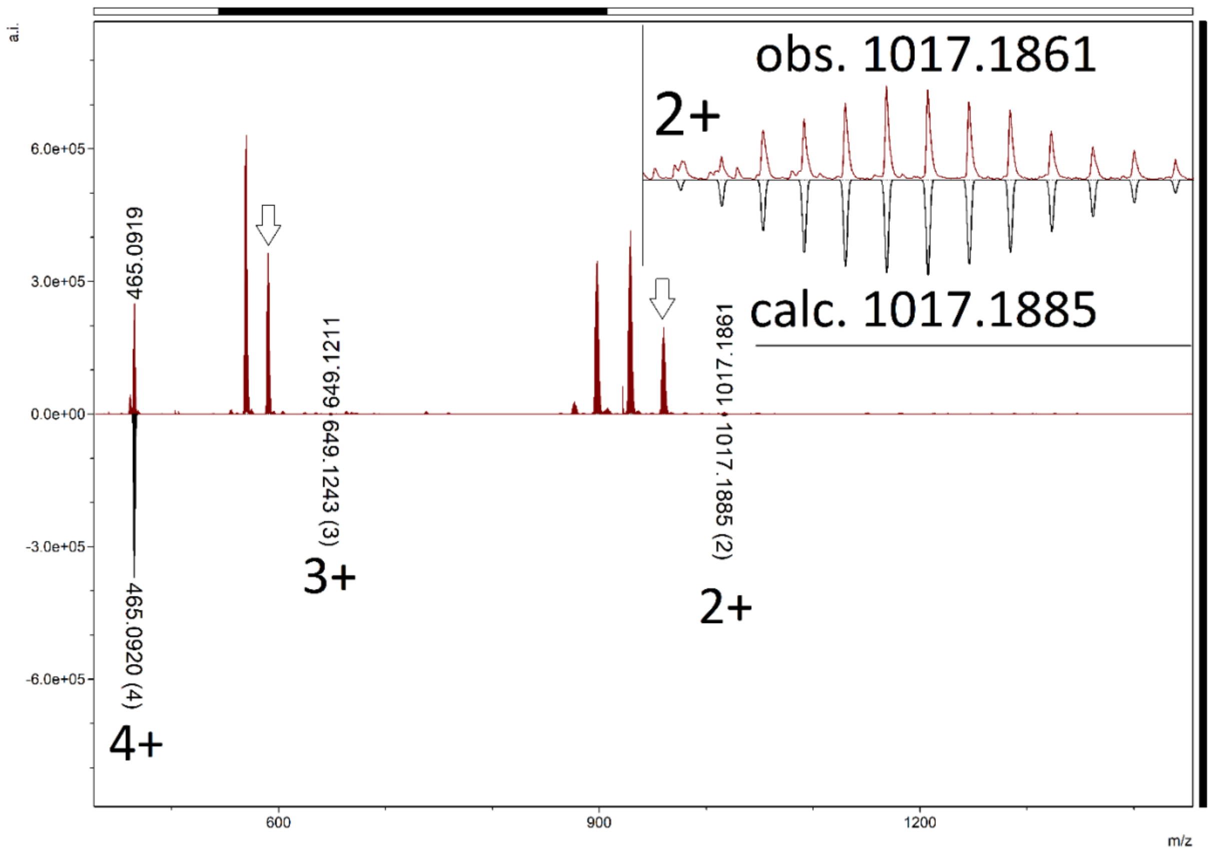

Supplementary Figure S38. Full HR-ESI-MS spectra of **DAQ⊂MOC** with annotation to different charged states of $[Pd_2L_4]^{n+}[OTf^-]_{4-n}$ for n= 1-4. Upper right: zoom in into 2+ species showing the measured value and isotopic pattern (top) and the calculated spectra (bottom). Black arrows indicate MOC without guest that appears due to ionization and dilution during MS measurements.

ESI-HRMS (m/z) calculated for $[(Pd_2L_4DAQ)(OTf)_2]^{2+}$ = 1017.1885; found 1017.1861. $[(Pd_2L_4DAQ)(OTf)_1]^{3+}$ = 649.1243; found 649.1211. $[(Pd_2L_4DAQ)(OTf)_0]^{4+}$ = 465.0920; found 465.0919.

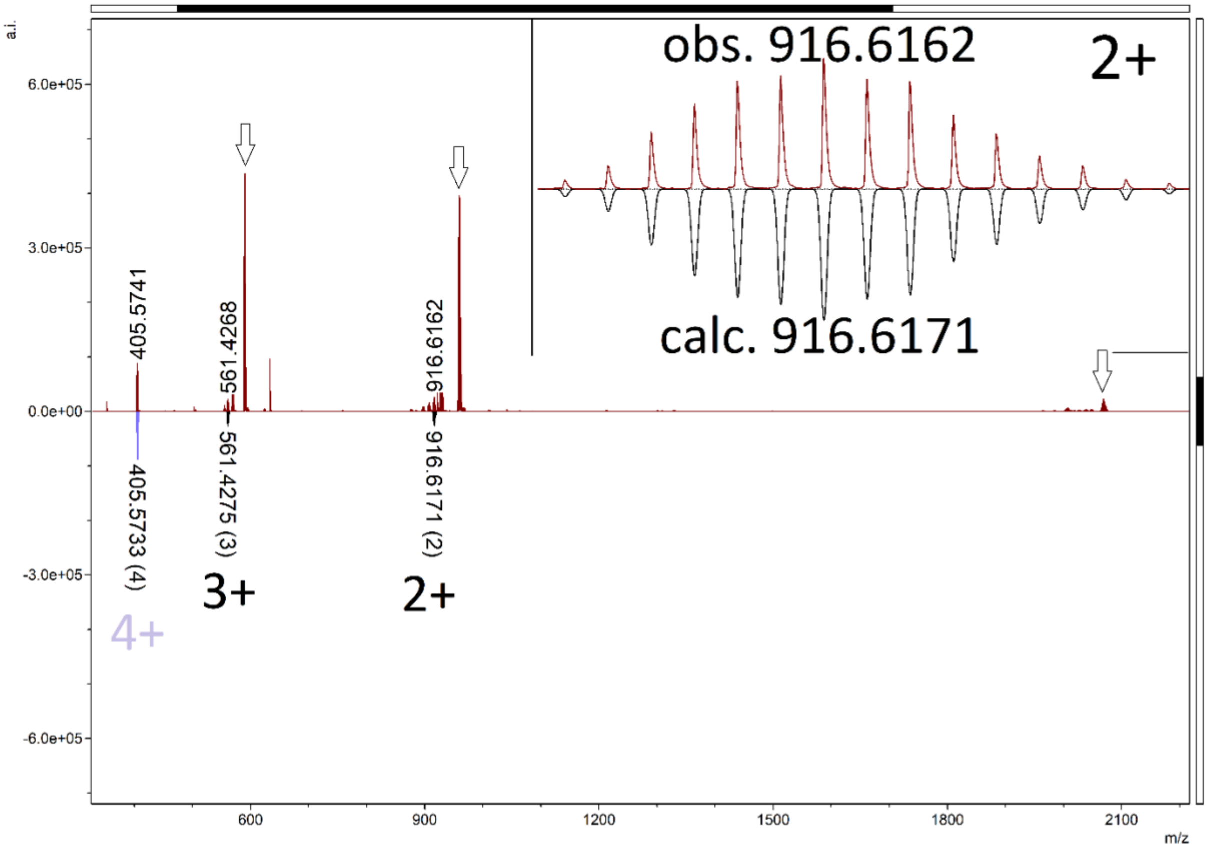

Supplementary Figure S39. Full HR-ESI-MS spectra of **NO_3⊂MOC** with annotation to different charged states of $[Pd_2L_4]^{n+}[OTf^-]_{3-n}[NO_3]_1$ for n= 1-3. Upper right: zoom in into 2+ species showing the measured value and isotopic pattern (top) and the calculated spectra (bottom). Black arrows indicate MOC with only $^-$OTf counterions; violet value belongs to NO_3 or $^-$OTf related species as all counterions are removed.

ESI-HRMS (m/z) calculated for $[(Pd_2L_4DAQ)(OTf)_1(NO_3)_1]^{2+}$ = 916.6171; found 916.6162. $[(Pd_2L_4DAQ)(OTf)_0(NO_3)_1]^{3+}$ = 561.4275; found 561.4268. $[(Pd_2L_4DAQ)(OTf)_0(NO_3)_0]^{4+}$ = 405.5733; found 405.5741.

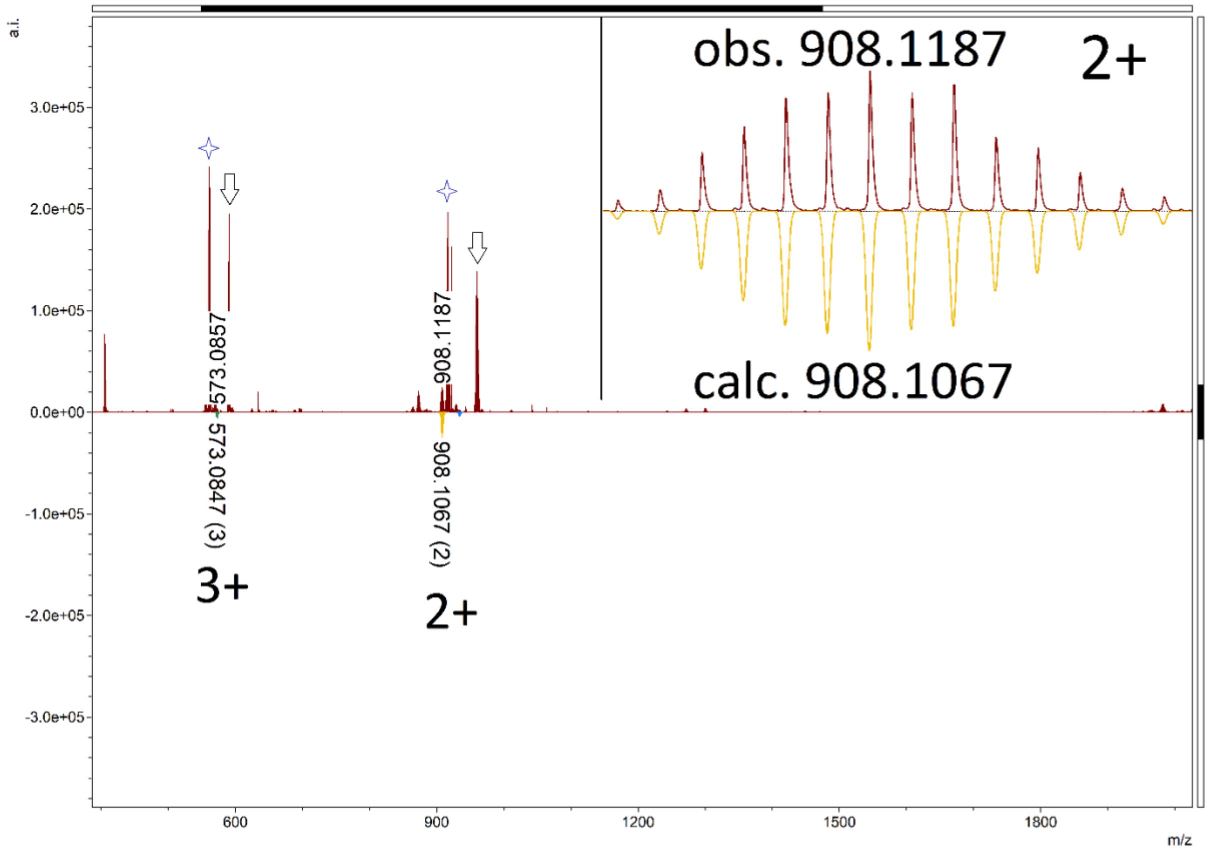

Supplementary Figure S40. Full HR-ESI-MS spectra of **HSO_4⊂MOC** with annotation to different charged states of $[Pd_2L_4]^{n+}[OTf^-]_{3-n}[HSO_4]_m$ for n= 1-3. Upper right: zoom in into 2+ species showing the measured value and isotopic pattern (top) and the calculated spectra (bottom). Black arrows indicate MOC with only $^-$OTf counterions; blue stars mark species with NO_3 counterions (likely due to impurity in MS chamber).

ESI-HRMS (m/z) calculated for $[(Pd_2L_4)(OTf)_1(HSO_4)_1]^{2+}$ = 934.1029; found 934.1019. $[(Pd_2L_4)(OTf)_0(HSO_4)_2]^{2+}$ = 908.1067; found 908.1187. $[(Pd_2L_4(OTf)_0(HSO_4)_1]^{3+}$ = 573.0847; found 573.0857.

Single Crystal X-Ray Diffraction Studies

Crystal Growth Conditions

All diffraction quality crystals were grown *via* slow vapor diffusion of diethyl ether into acetonitrile solutions of MOCs. Acetonitrile solutions of MOCs were prepared analogously to DMSO solutions as described in the *General procedure for MOC synthesis* section of the Supporting Information. Although guest binding of **HSO_4** and **NO_3** could be observed in acetonitrile, attempts to grow crystals when these guests were introduced as tetrabutylammonium salts were unsuccessful. Instead, **NO_3** was introduced via sub stoichiometric incorporation of $Pd(NO_3)_2(H_2O)_2$ in place of a fraction of the required $[Pd\text{-}(MeCN)_4][OTf]_2$, and **$HSO_4$** as an impurity in sodium 3-nitrobenzenesulfonate, itself crystalizing with **MOC**, and was found to reside within the MOC cavity upon crystallization. In detail, 1 mL to 3 mL of a MOC solution in acetonitrile (1mM MOC concentration) in a 4 mL vial was placed within a 20 mL vial. Then, approximately 5mL of diethyl ether was placed around the 4 mL vial. The 20 mL vial was capped and sealed with parafilm and allowed to sit for up to 7 days. We note that **DAQ⊂MOC** began to form crystals within 24 hours (as compared to over the course of a week for the other MOCs), presumably due to the rigid structure and slow guest exchange within the MOC cavity.

Crystallography Methods

Low-temperature (100 K) diffraction data (φ- and ω-scans) were collected on a Bruker X8 Kappa Duo four-circle diffractometer coupled to a Smart Apex2 CCD detector, with Mo Kα radiation (λ = 0.71073 Å) from an IμS micro-source. The diffractometer was purchased with the help of funding from the National Science Foundation (NSF) under Grant Number CHE0946721. The structure was solved by direct methods using SHELXSS4 and refined against F2 on all data by full-matrix least squares with SHELXL-97S5 following established refinement strategies (Müller, P. Practical suggestions for better crystal structures. Crystallogr. Rev. 15, 57-83 (2009)).

Crystal Structure Analysis

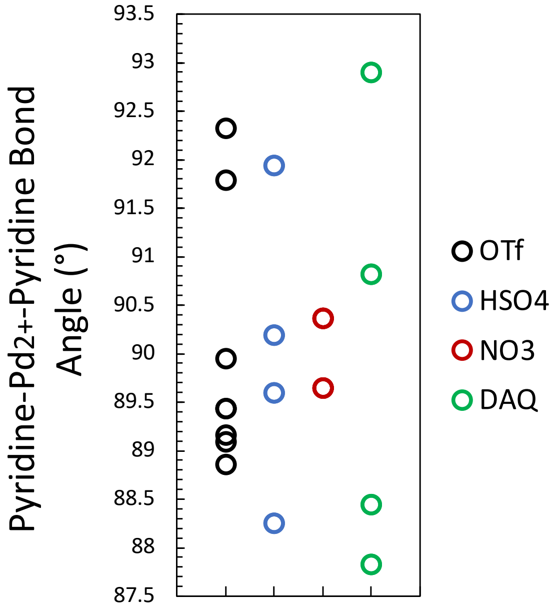

Supplementary Figure S41. Observed Pyridine-Pd^{2+}-Pyridine bond angles for the four crystal structures of host-guest complexes obtained in this study. Each **HSO_4** data point appears as two overlapping measurements, and each **NO_3** data point appears as four overlapping measurements.

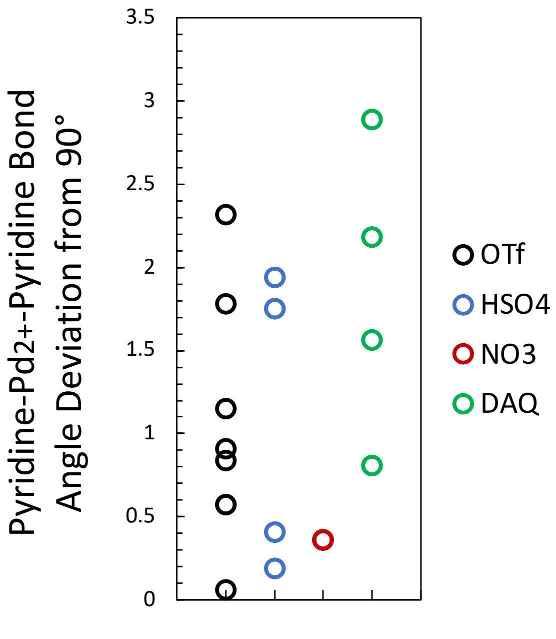

Supplementary Figure S42. Observed Pyridine-Pd^{2+}-Pyridine bond angle deviations from 90° (based on the bond angles reported in the previous figure) for the four crystal structures of host-guest complexes obtained in this study. Each **HSO_4** data point appears as two overlapping measurements, and each **NO_3** data point appears as four overlapping measurements.

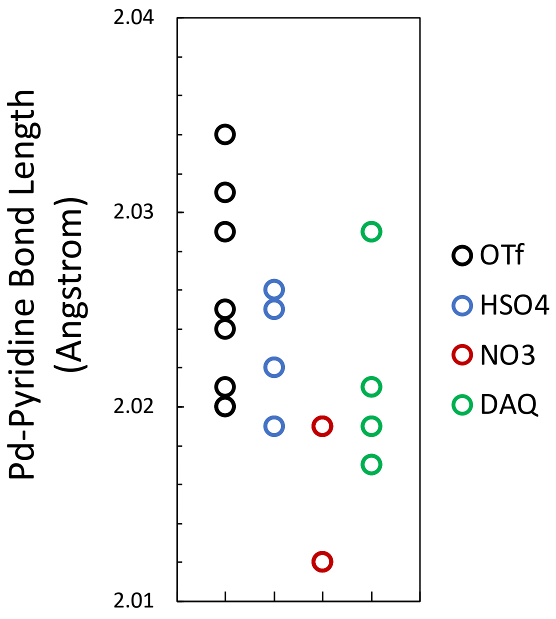

Supplementary Figure S43. Observed Pyridine-Pd^{2+} bond lengths for the four crystal structures of host-guest complexes obtained in this study. Each **HSO_4** data point appears as two overlapping measurements, and each **NO_3** data point appears as four overlapping measurements.

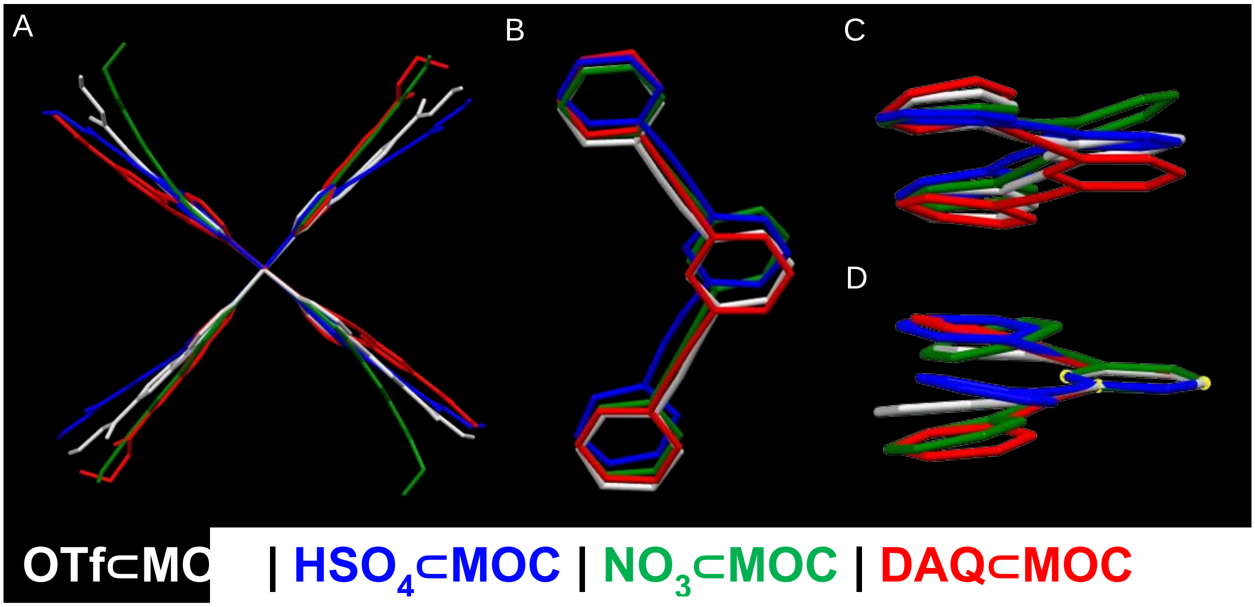

Supplementary Figure S44. Crystal structure overlays of all MOC host-guest complexes (guests and hydrogen atoms omitted for clarity). Structures are stacked over both Pd^{2+} atoms and a pyridine nitrogen atom (A, B, and C). **A.** Full cage stack looking down the Pd^{2+}-Pd^{2+} axis. **B.** Subsection of the stack in panel A showing only a single bis-pyridine ligand from each cage. **C.** Adjusted view of the stack in panel B to highlight the distortion of the bis-pyridine ligands. **D.** A modified stack of panel C where structures were aligned on the center aryl ring of the bis-pyridine ligands (stacking atoms are selected in the image).

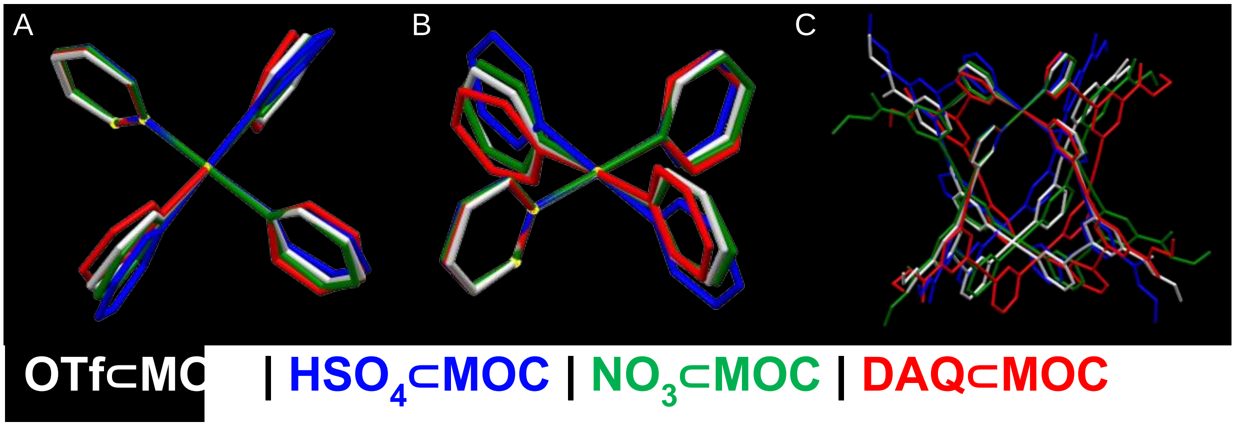

Supplementary Figure S45. Crystal structure overlays of all MOC host-guest complexes (guests and hydrogen atoms omitted for clarity). Structures are stacked over one Pd^{2+} atom, an adjacent pyridine nitrogen atom, and an adjacent carbon atom to that nitrogen (highlighted in panel A). **A.** Top-down view of Pd^{2+}-pyridine coordination motif of the host-guest complexes. **B.** A shallower view of the stack in panel A to better highlight relative pyridine moiety positions. **C.** Full cage stack illustrating the off-axis positioning of the Pd^{2+}-pyridine coordination structures relative to the Pd^{2+}-Pd^{2+} axis (as shown in Supplementary Figure S39A).

Thermal Ellipsoid Plots of Crystal Structures

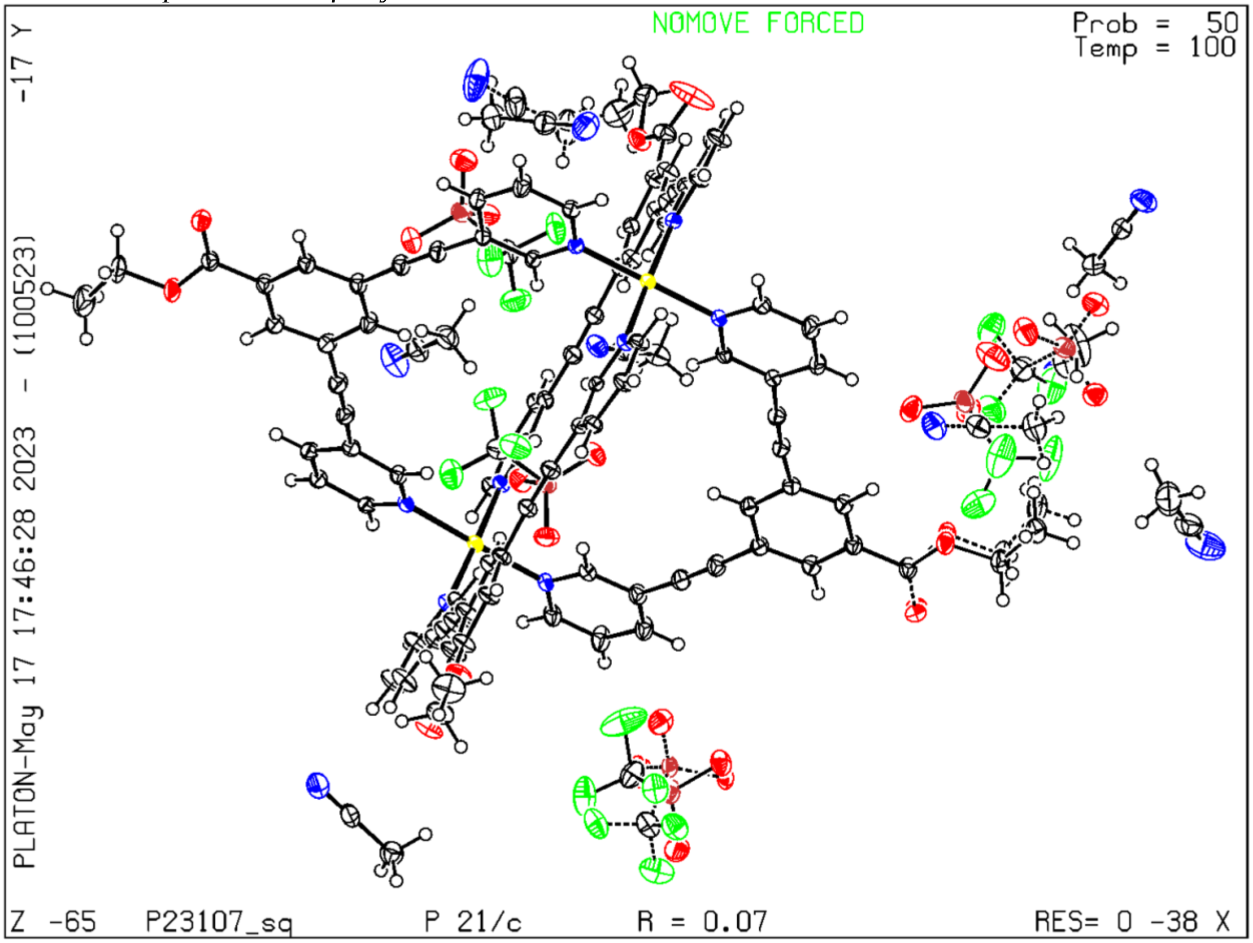

Supplementary Figure S46. Thermal ellipsoid plot for the **OTf⊂MOC** drawn at a 50% probability.

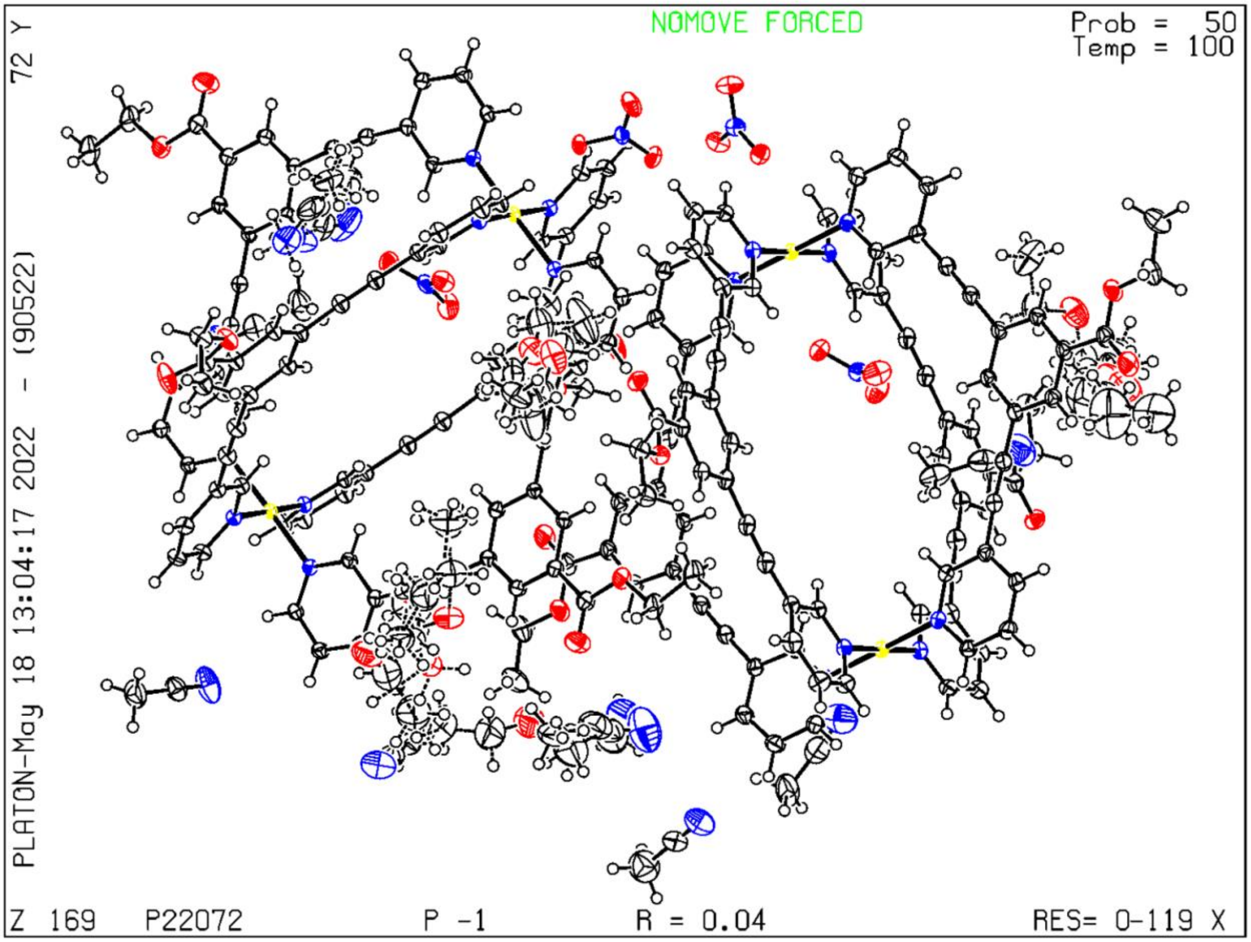

Supplementary Figure S47. Thermal ellipsoid plot for the **NO_3⊂MOC** drawn at a 50% probability.

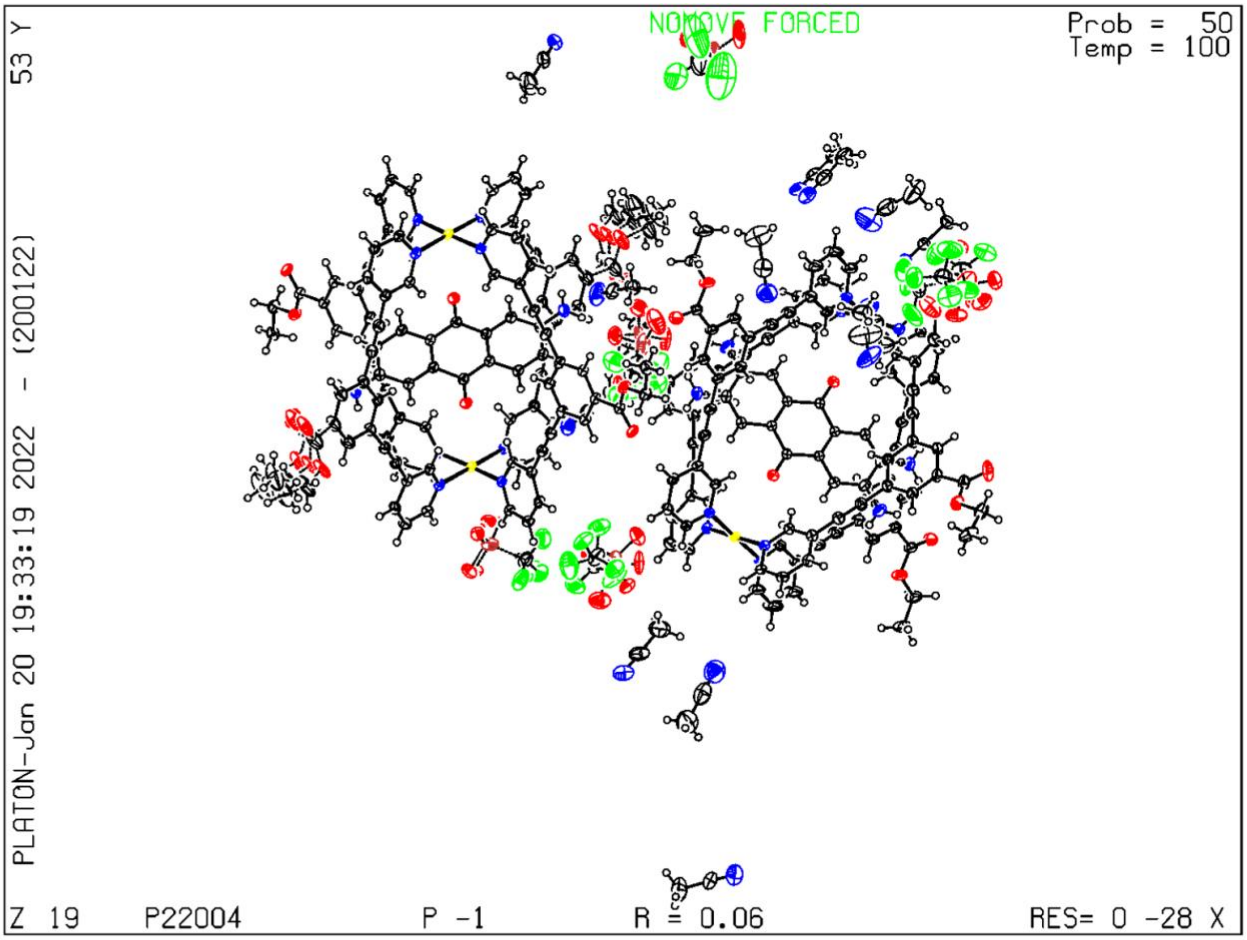

Supplementary Figure S48. Thermal ellipsoid plot for the **DAQ⊂MOC** drawn at a 50% probability.

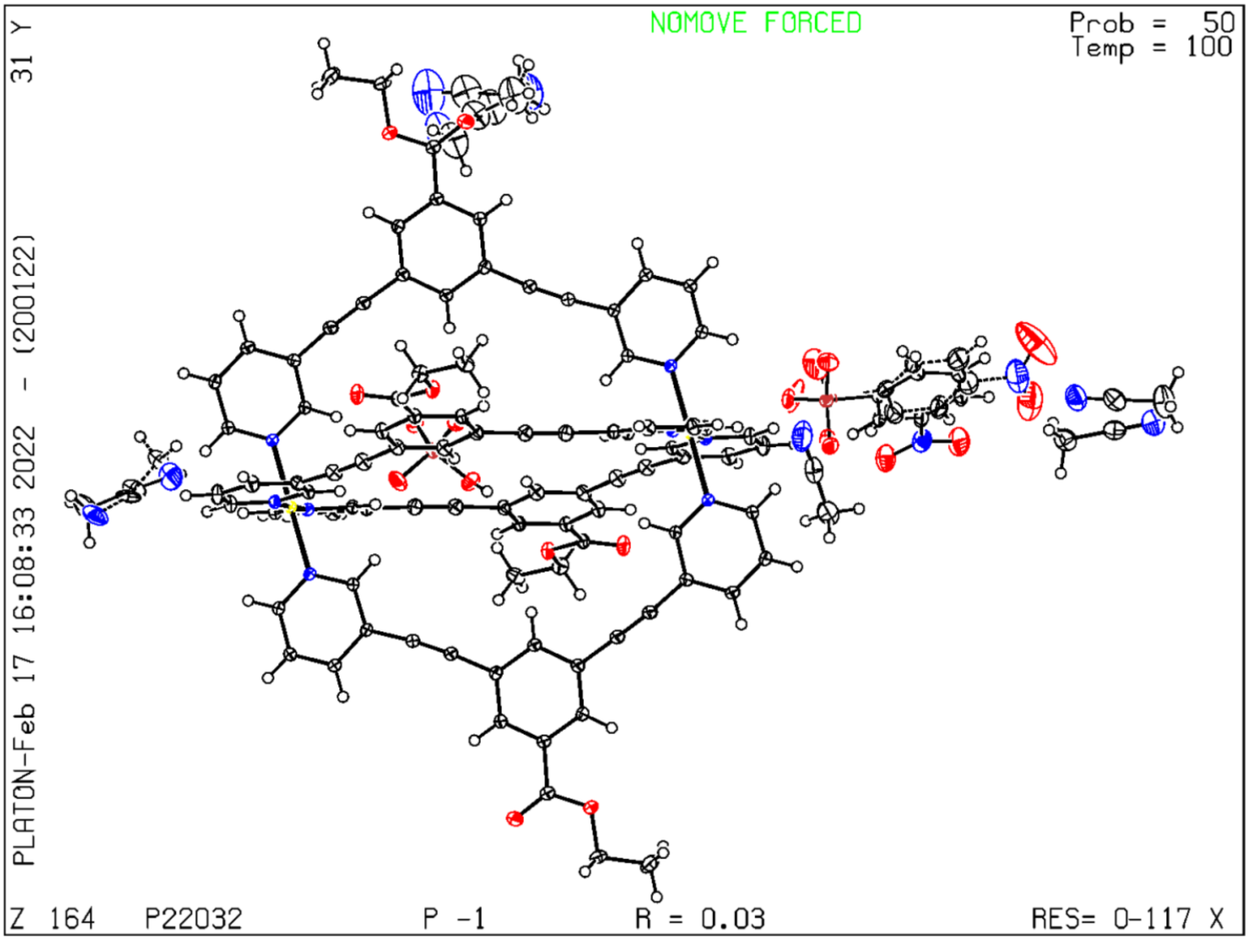

Supplementary Figure S49. Thermal ellipsoid plot for the **HSO_4⊂MOC** drawn at a 50% probability.

Supplementary Note DFT Calculations

A Triflate-bound cage structure was prepared from an experimental crystal structure in which the cage was already bound to a triflate ion. The structure was manually cleaned to remove any loose solvents, and the triflate-free cage was also generated by removing the triflate ion. We employed a developer version of the GPU-accelerated TeraChem v1.9[1] code to carry out DFT calculations. All calculations involved initial geometry optimization and vibrational numerical frequency calculations, followed by a higher-level single-point calculation using an implicit solvent. These calculations were performed using the B3LYP global hybrid[2] functional with a semi-empirical dispersion (i.e., DFT-D3)[3] correction with BJ damping[4]. Geometry optimization and thermochemistry calculations employed the LACVP* basis set consisting of LANDL2Z[5] effective core potential for Pd and 6-31G* basis for all other atoms. Geometry optimizations were carried out using the L-BFGS algorithm. Default tolerances of 4.5×10^{-4} hartree/bohr and 10^{-6} hartree were applied in the convergence criteria for the gradient and energy difference between steps, respectively. Single point energy calculations of optimized structures were carried out using the B3LYP-D3(BJ) functional, LANDL2Z effective core potential for Pd, 6-311G** basis for all other atoms, and an accelerated implementation of the C-PCM model[6] using a dielectric of $\varepsilon = 46.7$ to model DMSO solvent.

To contextualize the reported OTf binding energy referenced in the main text, an additional calculation was performed to determine the guest displacement enthalpy (ΔG) relative to the OTf-bound cage for **DAQ**. A value of -17.5 kcal/mol was found, suggesting an enthalpic preference for **DAQ** binding which is qualitatively consistent with experimental observations, although quantitatively represents a large overestimate of the observed binding free energy ($\Delta G = -6.4\ kcal/mol$). We hypothesize that this discrepancy is either due to the implicit solvent treatment of our calculation, as we are only accounting for electrostatic interactions between and solvent and solutes, or the fact that these calculations do not consider any secondary potential interactions outside the cage. It has been recently reported that weak, non-covalent binding to the exterior surfaces (interactions with protons H_b, H_c, H_d as labeled in Figure 2A) of an isostructural Pd_2L_4 MOC can reduce the binding affinity for quinone-type guests within the cavity of this structure.[7] We speculate that **OTf** displaced from the interior of the MOC upon **DAQ** binding may participate in such an interaction, as evidenced by the slight down-field shift for the resonances associated with H_b upon **DAQ** binding (see Figure 2C).

Supplementary References

(1) Seritan, S.; Bannwarth, C.; Fales, B. S.; Hohenstein, E. G.; Isborn, C. M.; Kokkila-Schumacher, S. I. L.; Li, X.; Liu, F.; Luehr, N.; Snyder Jr, J. W.; et al. TeraChem: A graphical processing unit-accelerated electronic structure package for large-scale ab initio molecular dynamics. *WIREs Computational Molecular Science* **2021**, *11* (2), e1494. DOI: https://doi.org/10.1002/wcms.1494 (acccessed 2023/08/18).

(2) Becke, A. D. Density-functional thermochemistry. iii. The role of exact exchange. *Journal of Chemical Physics* **1993**, *98* (7), 5648-5652. Lee, C.; Yang, W.; Parr, R. G. Development of the Colle-Salvetti correlation-energy formula into a functional of the electron density. *Physical Review B* **1988**, *37*, 785--789. (acccessed Jan). Stephens, P. J.; Devlin, F. J.; Chabalowski, C. F.; Frisch, M. J. Ab Initio Calculation of Vibrational Absorption and Circular Dichroism Spectra Using Density Functional Force Fields. *The Journal of Physical Chemistry* **1994**, *98* (45), 11623-11627.

(3) Grimme, S.; Antony, J.; Ehrlich, S.; Krieg, H. A consistent and accurate ab initio parametrization of density functional dispersion correction (DFT-D) for the 94 elements H-Pu. *The Journal of chemical physics* **2010**, *132* (15), 154104.
(4) Grimme, S.; Ehrlich, S.; Goerigk, L. Effect of the damping function in dispersion corrected density functional theory. *Journal of Computational Chemistry* **2011**, *32* (7), 1456-1465. DOI: https://doi.org/10.1002/jcc.21759 (acccessed 2023/08/14).
(5) Hay, P. J.; Wadt, W. R. Ab initio effective core potentials for molecular calculations. Potentials for the transition metal atoms Sc to Hg. *The Journal of Chemical Physics* **1985**, *82* (1), 270-283.
(6) Liu, F.; Luehr, N.; Kulik, H. J.; Martínez, T. J. Quantum Chemistry for Solvated Molecules on Graphical Processing Units Using Polarizable Continuum Models. *Journal of Chemical Theory and Computation* **2015**, *11* (7), 3131-3144.
(7) Marti-Centelles, V.; Spicer, R. L.; Lusby, P. J. *Chem. Sci.* **2020**, 11, 3236-3240.
